# Synthesis and Evaluation of Some New Aza-*B*-homocholestane Derivatives as Anticancer Agents

**DOI:** 10.3390/md12041715

**Published:** 2014-03-25

**Authors:** Yanmin Huang, Jianguo Cui, Sijing Chen, Qifu Lin, Huacan Song, Chunfang Gan, Bin Su, Aimin Zhou

**Affiliations:** 1 Key Laboratory of Beibu Gulf Environment Change and Resources Utilization, College of Chemistry and Life Science, Guangxi Teachers Education University, Nanning 530001, China; E-Mails: huangyanmin828@163.com (Y.H.); huaxue37z@126.com (S.C.); linqifu335@163.com (Q.L.); ganchunfang2008@126.com (C.G.); 2 School of Chemistry and Chemical Engineering, Sun Yat-sen University, Guangzhou 510275, China; E-Mail: YjhXhc@mail.sysu.edu.cn; 3 Clinical Chemistry Program, Department of Chemistry, SI 424, Cleveland State University, Cleveland, OH 44115, USA; E-Mails: b.su@csuohio.edu (B.S.); a.zhou@csuohio.edu (A.Z.)

**Keywords:** aza-*B*-homo-cholestane derivatives, anticancer agents, cytotoxicity, 3D multicellular spheroids screening, apoptosis

## Abstract

Using analogues of some marine steroidal oximes as precursors, a series of aza-*B*-homocholestane derivatives possessing different substituted groups at the 3-position of the steroidal nucleus were synthesized. Their biological activity against cancer cell proliferation was determined with multiple cancer cell lines. Aza-*B*-homocholestane derivatives possessing 3-hydroxyl, 3-hydroximino and 3-thiosemicarbazone groups displayed remarkable cytotoxicity to cancer cells via apoptosis inducing mechanism. Compounds **5**, **10**, **12**, **15** and **18 **exhibited better potency to inhibit cancer cell proliferation. In addition, compound 15 was further evaluated with three dimensional (3D) multicellular spheroids assay to determine its potency against spheroid growth. The structure-activity relationship (SAR) generated in the studies is valuable for the design of novel chemotherapeutic agents.

## 1. Introduction

**Figure 1:**
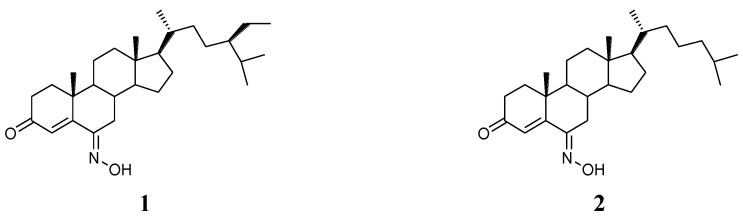
Chemical structures of compounds **1 **and **2**.

Natural products derived from marine organisms are important resources of biologically active compounds. Compounds with steroidal skeletons are abundant in marine sponges [1]. However, marine steroids having an oxime group have been less reported in the literatures. (6*E*)-Hydroximino-24-ethylcholest-4-en-3-one (**1**) and (6*E*)-hydroximinocholest-4-en-3-one (**2**) were two interesting steroidal compounds having 6-oxime group, and were isolated from the sponges *Cinachyrella* (*C. alloclada* and *C. apion*) (Figure 1) [2]. Compound **2** showed cytotoxicity with IC_50_ of 1.25 μg/mL against P-388, A-549, HT-29 cells, and 2.5 μg/mL toward MEL-28 cells. Based on the results, different types of steroidal oxime derivatives were synthesized and their cytotoxicities were evaluated [3,4,5,6,7,8]. 

Aza-homosteroids are a class of steroid compounds that were synthesized and modified in order to increase the biological activity of the lead steroids. The synthesis of some aza-homosteroid compounds with unusual and interesting structures had been reported recently [9,10,11,12,13,14]. These compounds exhibited cytotoxic, antibacterial, antileukemic, and antiandrogenic activities. Studies of aza-homosteroids revealed that the presence of the characteristic group (-NH-CO-) in the aza-homosteroid molecule is important in lowering the acute toxicity and improving anticancer activity of the compounds [15,16].

In our previous work, a series of 3-aza-*A*-homo and 4-aza-*A*-homo steroidal lactams were prepared and evaluated against the proliferation of SGC 7901 (human ventriculi carcinoma cell line), HeLa (human cervical carcinoma cell line) and Bel 7404 (human liver carcinoma cell line) [17,18,19]. The results showed that some of these steroidal lactams significantly inhibited the proliferation of the tumor cell lines tested and induced cancer cell apoptosis as well. In order to find more active derivatives as potential antitumor agents, a series of the new aza-*B*-homecholestane derivatives possessing different aza-position and various substitute groups on the 3-position of steroidal nucleus were designed and synthesized taking analogues of compound **2** as precursors. The antiproliferative activities of these new compounds were evaluated with different cancer cell lines.

## 2. Results and Discussion

### 2.1. Chemistry

**Scheme 1:**
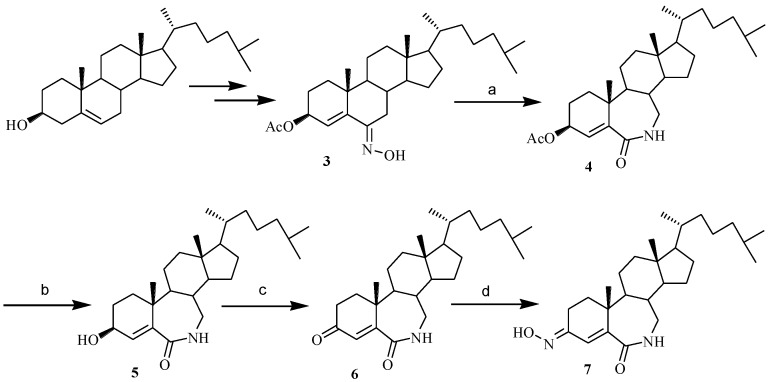
Synthesis of 3-hydroximino-7-aza-*B*-homocholest-4-en-6-one.

First, 3-hydroximino-7-aza-*B*-homocholest-4-en-6-one (**7**) was synthesized as listed in Scheme 1. The synthesis of steroidal oxime (**3**) had been reported by our group [7]. Beckman rearrangement of **3** in SOCl_2_/THF gave 3-acetoxy-7-aza-*B*-homocholest-4-en-6-one (**4**). The structure of **4** was confirmed by ^1^H NMR chemical shifts of 7a-protons at 3.16–3.21 ppm (2H, m). Compound **5** was obtained by deacetylation of **4** in aqueous solution of 13% K_2_CO_3_. The oxidation of compound **5** with Jones reagent afforded 7-aza-*B*-homocholest-4-en-3,6-dione (**6**). Last, the oxime **7** was produced in a yield of 42% by the reaction of **6** with hydroxylamine hydrochloride in ethanol in the presence of NaOAc. The structure of **7** was confirmed by analysis of the proton and carbon NMR chemical shifts. The downfield chemical shift of 2β-H at 3.453 ppm (dd, *J* = 15.3 and 4.8 Hz, due to the deshielding influence of the OH in the hydroximino group) and C-3 at 158.6 ppm demonstrated the (*E*)-configuration and formation of 3-hydroximino in compound **7**.

Reagents and conditions: (a) SOCl_2_/THF, 0 °C; (b) 13% K_2_CO_3_/CH_3_OH, stirred, 4 h; (c) Jones reagent/acetone, r. t., 2 h; (d) H_2_NOH.HCl/Na_2_Ac.3H_2_O/EtOH, reflux.

**Scheme 2:**
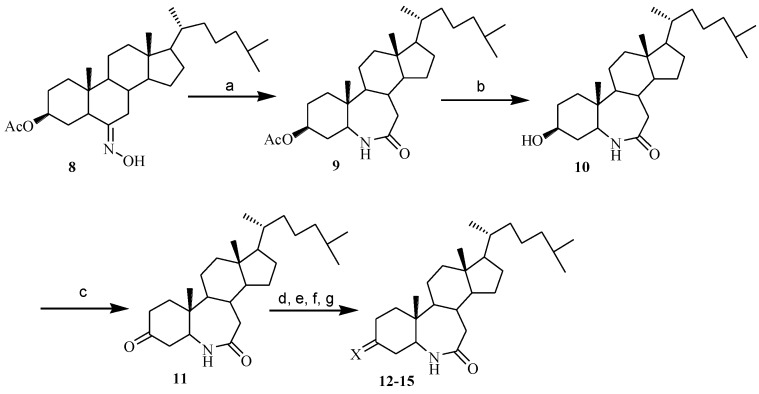
Synthesis of compounds **9**–**15**.

**12**: X = NOH; **13**: X = NOCH_3_; **14**: X = NOCH_2_Ph; **15**: X = NNHCSNH_2_. Reagents and conditions: (a) SOCl_2_/THF, 0 °C; (b) 13% K_2_CO_3_/CH_3_OH, reflux, 4 h; (c) Jones reagent/acetone, r. t., 2 h; (d) H_2_NOH.HCl/Na_2_Ac.3H_2_O/EtOH, reflux; (e) CH_3_ONH_2_.HCl/95% C_2_H_5_OH, 60 °C; (f) PhCH_2_ONH_2_/95% C_2_H_5_OH, 60 °C; (g) H_2_NC(S)NHNH_2_/EtOH, 60 °C.

Similarly, compounds **12**–**15** possessing the 3-substituted-6-aza-*B*-homocholest-7-one key feature in their structures were synthesized with 8 steps using cholesterol as starting material (Scheme 2). Compound **8** was synthesized based on the known methodology [8]. The structure of **9** was affirmed by proton and carbon NMR spectrums. In the ^1^H NMR spectrum, the resonances showing of C_5_-αH at 3.47–3.40 ppm (1H, m), C_7a_-H at 2.36–2.22 ppm (2H, m) and 7-C at 176.2 ppm demonstrated a formation of NH-CO- bond and a position of 6-NH. The oxidization of **10** by Jones reagent generated compound **11**. The reaction of compound **11 **with HONH_2_·HCl, CH_3_ONH_2_·HCl, PhCH_2_ONH_2_·HCl or thiosemicarbazide afforded the corresponding products **12**–**15**. Their structures were confirmed by IR and NMR spectrum, and the mixture of *E*- and *Z*-stereoisomer was obtained in preparation of **13**–**15**, respectively.

**Scheme 3:**
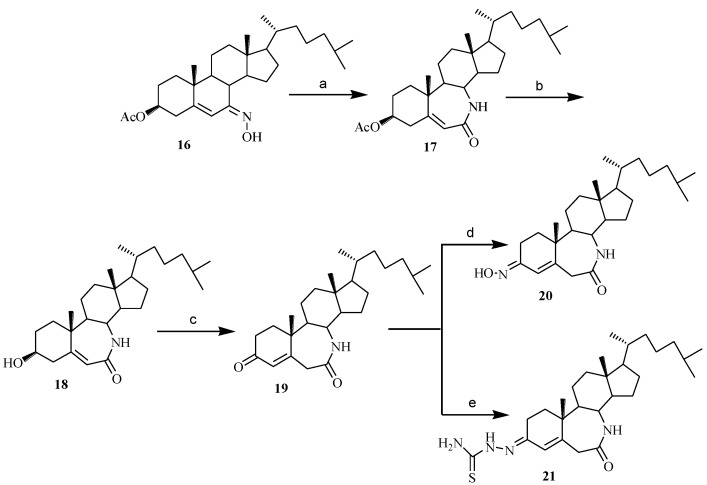
Synthesis of compounds **17**–**21**.

Last, in order to determine if the aza-position has an effect on the compounds’ cytotoxicity, compounds **17**–**21** possessing the structure of 7a-aza-*B*-homocholest-4(or 5)-ene were prepared (Scheme 3). Beckman rearrangement of **16** provided 3-acetoxy-7a-aza-*B*-homocholest-5-ene-7-one (**17**). The downfield chemical shift of C_8_-H at 3.292 ppm (1H, t, *J* = 8.4) in ^1^H NMR spectrum of **17** indicated the position of 7a-aza. In the oxidization of compound **18** by Jones reagent, 5,6-double bond of **18** was transformed into more stable 4,5-double bond of compound **19**. Further, compounds **20** and **21** were obtained by the reaction of **19** with HONH_2_·HCl and thiosemicarbazide respectively. The structures of **20**, **21 **were confirmed with proton and carbon NMR.

Reagents and conditions: (a) SOCl_2_/THF, 0 °C; (b) 13% K_2_CO_3_/CH_3_OH, reflux, 4 h; (c) Jones reagent/acetone, r.t., 2 h; (d) H_2_NOH.HCl/Na_2_Ac.3H_2_O/EtOH, reflux; (e) H_2_NC(S)NHNH_2_/EtOH, 60 °C.

### 2.2. Biological Evaluation

#### 2.2.1. Cytotoxicity Determination

All compounds were evaluated for their antiproliferative activities against SGC 7901 (human gastric carcinoma), HeLa (human cervical carcinoma), Bel 7404 (human liver carcinoma) GNE 2 (nasopharyngeal carcinoma), SPC-A (lung carcinoma) and Tu 686 (laryngocarcinoma) cell lines using the MTT assay. The results were summarized as IC_50_ values in µM in Table 1.

**Table 1:** *In vitro* ^a^ antiproliferative activities of aza-*B*-homocholestane derivatives (IC_50_ in µM).

Compounds	SGC 7901	HeLa	Bel 7404	GNE 2	SPC-A	Tu 686
**4**	91.8	20.8	14.2	34.5	92.5	85.4
**5**	13.2	26.5	9.8	7.2	7.5	25.4
**6**	>100	31.4	>100	29.2	>100	58.6
**7**	>100	9.3	58.4	>100	>100	98.5
**8**	25.0	52.1	17.4	34.8	15.6	59.8
**9**	66.3	8.3	15.4	15.2	98.5	58.4
**10**	31.8	3.2	11.6	9.5	47.6	42.5
**11**	90.4	8.7	8.7	16.1	74.5	6.8
**12**	92.4	9.1	31.3	11.3	34.8	28.5
**13**	70.5	35.8	69.8	63.8	78.7	36.0
**14**	26.5	22.9	37.8	5.4	34.0	23.6
**15**	29.5	5.5	9.8	12.4	6.6	28.9
**16**	>100	>100	>100	>100	48.5	>100
**17**	>100	>100	>100	17.5	>100	36.2
**18**	11.3	6.0	4.8	20.1	23.4	25.3
**20**	25.7	35.7	42.2	43.0	35.6	42.5
**21**	24.6	16.4	45.2	39.0	ND ^b^	ND
**Cisplatin**	6.7	10.1	23.2	16.8	15.2	17.5

^a^ Data represent the mean values of three independent determinations; ^b^ Not determined.

As shown in Table 1, compounds **8**–**15** possessing the structure of 3-substituted-6-aza-*B*-homocholest-7-one had better antiproliferative activity than compounds **4**–**7** with the structure of 3-substituted-7-aza-*B*-homocholest-4-ene-6-one and compounds **17**, **18**, **20**, **21** with the structure of 3-substituted-7a-aza-*B*-homocholest-4(or 5)-ene-7-one. Compound **5** with 3-hydroxyl group exhibited better antiproliferative activity than the compound **4** with 3-acetoxyl, the **6** with 3-carbonyl and the **7** with 3-hydroximino groups. Comparing to analogues **8**–**15**, compounds **10**, **12**, **15** possessing 3-hydroxyl, 3-hydroximino and 3-thiosemicarbazone groups displayed better cytotoxicity. Moreover, compound **18** with 3-hydroxyl group showed the best antiproliferative activity in analogues **17**, **18** and **20**, **21**. 

Interestingly, all compounds with 3-hydroxyl, hydroximino and thiosemicarbazone groups exhibited a higher cytotoxicity against HeLa cells. For example, compound **10** with 3-hydroxyl and compound **15** with 3-thiosemicarbazone exhibited IC_50_ values of 3.2 and 5.5 µM to HeLa cells, respectively. Apparently, all compounds (**5**, **10**, **18**) bearing the same 3-hydroxyl and a different aza-position displayed a similar cytotoxicity against these cancer cells. However, compound **15** having same 3-thiosemicarbazone and 6-aza-*B*-homocholest-7-one key features in its structure showed a better antiproliferative activity to these cancer cells than compound **21** with the structure of 7a-aza-*B*-homocholest-5-ene-7-one except SGC 7901 cell.

Above results showed that the conversion of a carbonyl group at 3-position to a hydroxyl, hydroximino, or thiosemicarbazone groups would result in a significant increase of the antiproliferative activity, suggesting the importance of these functional groups in the biological function of the compounds. Obviously, the substitution of the 3-hydroxyl group remarkably increased its cytotoxic activity against these cancer cells in comparison with the 3-acetoxyl group.

Overall, compounds **5**, **10**, **12**, **15** and **18 **were found to be the most potent compounds as anticancer agents, and they displayed a similar antiproliferative activity, when compared to cisplatin (a positive control).

#### 2.2.2. 3D Multicellular Spheroids Screening

Screening and initial characterization of anticancer drugs typically use monolayer cultures of tumor cells. However, such monolayer cultures do not represent the characteristics of threedimensional (3D) solid tumors. The multicellular tumor spheroid model has an intermediate complexity between *in vivo* tumors and *in vitro* monolayer cell cultures. Considering the complexity of the *in vivo* situation, it is not surprising that many drugs that are effective in a twodimensional cell culture will lose efficacy with the *in vivo* assays. Limited penetration of the drug into tumor cell masses is one main factor that will lead to poor drug efficacy in animal models. Testing the efficacy of the drug with tumor spheroids may help to predict their *in vivo* potency [20]. Herein, compound **15** was examined with the 3D spheroid growth assay.

Spheroids were photographed in an inverted phase contrast microscope. A micrometer scale was photographed at the same magnification, and spheroid size was determined and compared.

**Figure 2:**
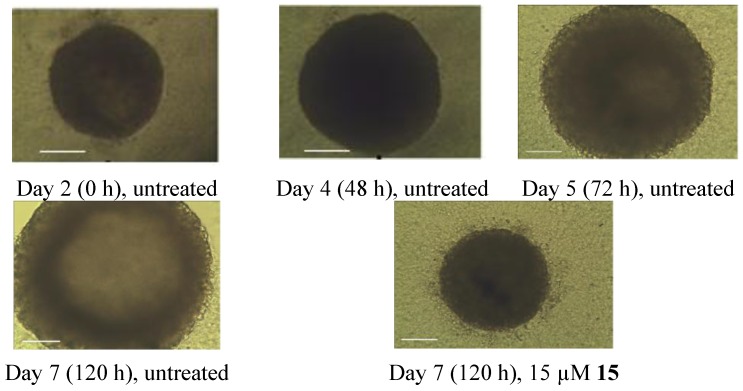
Time-lapse images of control untreated 5000-cell H-292 spheroid, and spheroid treated with 20 µM **15**. Scale bar is 100 µm.

In Figure 2, the spheroids treated with 15 µM of **15 **had a smaller size after 6 days than the untreated control spheroids. The result indicated that compound **15 **showed good tumor penetration ability. 

#### 2.2.3. Compound **5** Induced Apoptosis of Cancer Cells

**Figure 3:**
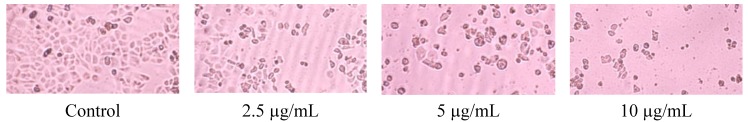
Photographs of the unstained cells were taken under Olympus model CKX31 at 100 magnification after treatment of MGC 7901 cells with various doses of compound **5** for 48 h.

To determine the molecular mechanism by which compound **5** inhibited cancer cell proliferation, we further analyzed the cytotoxicity of compound **5** in SGC-7901 cells. As shown in Figure 3, compound **5**-induced SGC-7901 cell death could be clearly observed. 

**Figure 4:**
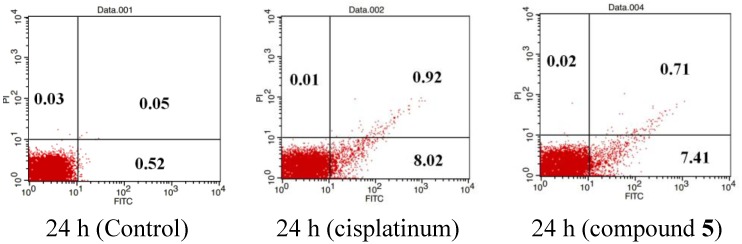
SGC-7901 cells were double-stained with annexin V/PI and analyzed by flow cytometry. Treatment with compound **5** (5 μg/mL) and cisplatinum (10 µg/mL) for 24 h induced apoptosis of SGC-7901 cells.

To determine whether the decreased viability of SGC-7901 cells was due to compound **5** induced apoptosis, the cells were treated with compound **5**, and Annexin V assay was performed. FITC-conjugated Annexin V is commonly used to determine apoptotic cells at an early stage. As shown in Figure 4, treatment with 5 μg/mL of compound **5** resulted in 8.12% PI/Annexin V double-labeled apoptotic cells (control: 0.57%; cisplatinum: 8.94%) after 24 h incubation (the lower right quadrant and the upper right quadrant which contains early and late apoptotic cells, respectively), suggesting compound **5** is a potent apoptotic inducer in gastric carcinoma cells. 

**Figure 5:**
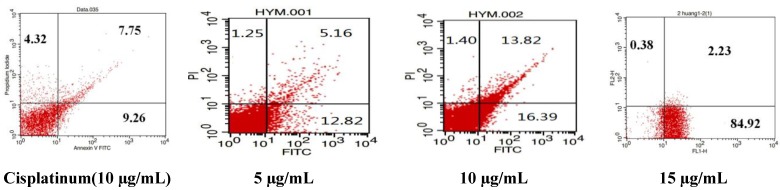
Dose depended apoptosis induced by compound **5** and cisplatinum (10 µg/mL) for 48 h.

The similar result was observed after treating with compound **5** in a dose dependent manner (Figure 5). Treatment with 5 μg/mL, 10 μg/mL, 15 μg/mL of compound **5** for 48 h resulted in 17.98, 30.21, 87.15% PI/Annexin V double-labeled apoptotic cells, proposing compound **5** induced the apoptosis of SGC-7901 cells further.

## 3. Experimental Section

### 3.1. Chemistry

The sterols were purchased from the Sinopharm Chemical Reagent Co., Ltd., Shanghai, China. All chemicals and solvents were analytical grade and solvents were purified by general methods before being used. Melting points were determined on an X4 apparatus and were uncorrected. Infrared spectra were measured with a Nicolet FT-360 Spectrophotometer (Thermo Fisher Scientific, New York, NY, USA). The ^1^H and ^13^C NMR spectra were recorded in CDCl_3_ on a Bruker AV-600 spectrometer (Bruker Corporation, Billerica, MA, USA) at working frequencies 600 and 150 MHz, and a Bruker AV-300 spectrometer at working frequencies 300 and 75 MHz, respectively. Chemical shifts are expressed in parts per million (δ) values and coupling constants (*J*) in Hertz. LREIMS were recorded on a Thermo-DSQ instrument (Thermo Fisher Scientific, New York, NY, USA), while HREIMS were measured on a Agilent 6210 TOFMS instrument (Agilent Technologies, Palo Alto, CA, USA). The cell proliferation assay was undertaken by a MTT method using 96-well plates on MLLTISKAN MK3 analysis spectrometer (Thermo Scientific, Shanghai, China). Annexin V assay was performed using FACS Calibur flow cytometry (Becton Dickinson, Biosciences, Franklin Lakes, NJ, USA).

Compounds **3**, **16** were prepared according to the procedures in the literature [7].

#### 3.1.1. 3-Acetoxy-7-aza-*B*-homocholest-4-ene-6-one (**4**)

The solution of thionyl chloride (1.5 mL) in 5 mL dry THF was added to a solution of the oxime **3** (300 mg) in dry THF (30 mL). The solution was stirred under anhydrous condition for 1 h at 0 °C. The reaction was terminated and water was added to the solution. The solution was neutralized with ammonia and the product was extracted with CH_2_Cl_2_ (20 × 3 mL). The combined extract was washed with water, 5% NaHCO_3_, and saturated brine, dried over anhydrous Na_2_SO_4_ and evaporated under reduced pressure to give a crude product which was chromatographed on silica gel (elution: Dichloromethane/methanol = 20/1) to give white solid 216 mg. Yield: 72%, mp 157–159 °C; IR (KBr) ν: 3195, 2938, 1740, 1659, 1470, 1377, 1230, 1136, 1230, 1058 cm^−1^; ^1^H NMR (CDCl_3_, 300MHz) δ: 0.70 (3H, s, 18-CH_3_), 0.86 (3H, d, *J* = 6.6, 26- or 27-CH_3_), 0.88 (3H, d, *J* = 6.6, 26- or 27-CH_3_), 0.91 (3H, d, *J* = 6.6, 21-CH_3_), 1.21 (3H, s, 19-CH_3_), 2.14 (3H, s, -OCOCH_3_), 3.16-3.21 (2H, m, C_7a_-H), 5.53 (1H, dd, *J* = 12.3, 4.5, C_3_-αH), 5.91 (1H, s, C_4_-H), 6.72 (1H, s, 6-NH); ^13^C NMR (CDCl_3_, 75MHz) δ: 169.9 (6-C), 169.8 (COCH_3_), 156.7 (5-C), 114.7 (4-C), 71.2 (3-C), 56.0 (17-C), 55.5 (14-C), 53.3 (9-C), 45.5 (7a-C), 42.9 (13-C), 42.6 (24-C), 39.6 (12-C), 39.5 (8-C), 36.6 (10-C), 36.1 (22-C), 35.7 (20-C), 34.6 (1-C), 28.1 (16-C), 28.0 (25-C), 24.1 (15-C), 23.8 (2-C), 22.8 (26 or 27-C), 22.5 (26 or 27-C), 21.8 (23-C), 21.4 (11-C), 21.3 (CH_3_CO), 20.8 (19-C), 18.6 (21-C), 12.0 (18-C); LRESI-MS(*m/z*): 458.3 [M + H]^+^ (Supplementary Information: Figure S1).

#### 3.1.2. 3-Hydroxy-7-aza-*B*-homocholest-4-ene-6-one (**5**)

20 mL of K_2_CO_3_ solution (13%) was added to a solution of compound **4** (1520 mg, 3.32 mmol) in CH_3_OH (200 mL). The reaction mixture was heated under reflux condition for 1 h. After completion of the reaction as indicated by TLC, the solvent was removed under reduced pressure. 200 mL of CH_2_Cl_2_ was added to dissolve solid and the resulting solution was washed with cold water and saturated brine. After drying over anhydrous sodium sulfate, the solvent was removed under reduced pressure, and the resulting crude product was purified by chromatography on silica gel using methanol/dichloromethane (30:1) as elution to give 988 mg of **5** as white solid. Yield: 65%, mp 233–234 °C; IR (KBr) ν: 3366, 2941, 2872, 1654, 1593, 1470, 1380, 1319, 1123, 898 cm^−1^; ^1^H NMR (CDCl_3_, 600MHz) δ: 0.71 (3H, s, 18-CH_3_), 0.88 (3H, d, *J* = 6.6, 26- or 27-CH_3_), 0.89 (3H, d, *J* = 6.6, 26- or 27-CH_3_), 0.92 (3H, d, *J* = 6.6, 21-CH_3_), 1.15 (3H, s, 19-CH_3_), 2.16-2.20 (1H, m, C_2_-H), 2.23 (1H, br s, -OH), 3.16-3.21 (1H, m, C_7a_-αH), 3.28-3.23 (1H, m, C_7a_-βH), 4.43 (1H, t, *J* = 5.4, C_3_-αH), 6.21 (1H, s, C_4_-H), 6.38 (1H, s, 7-NH); ^13^C NMR (CDCl_3,_ 75MHz) δ: 170.7 (6-C), 162.1 (5-C), 114.3 (4-C), 69.6 (3-C), 56.0 (17-C), 55.6 (14-C), 53.1 (9-C), 45.0 (7a-C), 42.7 (13-C), 42.6 (12-C), 39.6 (24-C), 39.5 (8-C), 36.7 (10-C), 36.1 (22-C), 35.7 (20-C), 34.7 (1-C), 28.2 (2-C), 28.1 (16-C), 28.0 (25-C), 24.2 (15-C), 23.8 (23-C), 22.8 (26 or 27-C), 22.6 (26 or 27-C), 21.8 (11-C), 21.7 (19-C), 18.6 (21-C), 12.0 (18-C); HRESI-MS(*m/z*): 416.3515 [M + H]^+^ (calcd for C_27_H_46_NO_2_, 416.3529) (Supplementary Information: Figure S2).

#### 3.1.3. 7-Aza-*B*-homocholest-4-ene-3,6-dione (**6**)

Jones reagent was added dropwise to the solution of **5 **(1 mmol) in 20 mL of acetone in 10 min at room temperature until the reaction solution didn’t fade. The mixture was stirred at room temperature for 1 h, and then neutralized with 10% K_2_CO_3_ solution. The suspension was poured over a silica gel column and eluted with ethyl acetate. The solvent was removed under reduced pressure. The residue was purified by chromatography on silica gel using methanol/dichloromethane (30:1) as elution to give 270 mg of **6** as white solid. Yield: 65%, mp 209–210 °C. IR (KBr) ν: 3509, 2933, 2868, 1695, 1658, 1605, 1462, 1376, 1249, 1074 cm^−1^; ^1^H NMR (CDCl_3_, 600MHz) δ: 0.72 (3H, s, 18-CH_3_), 0.88 (3H, d, *J* = 6.6, 26-CH_3_ or 27-CH_3_), 0.89 (3H, d, *J* = 6.6, 26 or 27-CH_3_), 0.93 (3H, d, *J* = 6.6, 21-CH_3_), 1.06 (3H, s, 19-CH_3_), 2.11-2.03 (2H, m, C_2_-H and C_1_-H), 2.62 (1H, dd, *J* = 15, 4.5, C_2_-H), 3.25-3.21 (1H, m, C_7a_-αH), 3.35-3.30 (1H, m, C_7a_-βH), 6.00 (1H, s, C_4_-H), 6.43 (1H, s, -NH); ^13^C NMR (75MHz, CDCl_3_) δ: 205.3 (3-C), 168.7 (6-C), 156.8 (5-C), 121.5 (4-C), 56.4 (17-C), 55.9 (14-C), 51.2 (9-C), 47.1(13-C), 45.9(7a-C), 42.7 (12-C), 40.4 (24-C), 39.5 (8-C), 39.3 (10-C), 36.9(22-C), 36.0 (20-C), 35.7 (1-C), 35.3 (2-C), 28.0 (16-C), 28.0 (25-C), 23.9 (15-C), 23.8 (23-C), 22.8 (26 or 27-C), 22.5 (26-C or 27-C), 21.9 (11-C), 20.5 (19-C), 18.6 (21-C), 12.0 (18-C); HRESI-MS (*m/z*): 414.3362 [M + H]^+^ (calcd for C_27_H_44_NO_2_, 414.3372) (Supplementary Information: Figure S3).

#### 3.1.4. 3-Hydroximino-7-aza-*B*-homocholest-4-ene-6-one (**7**)

CH_3_COONa.3H_2_O (120 mg, 0.33 mmol) and NH_2_OH.HCl (23 mg, 0.33 mmol) were added to the solution of 120 mg (0.27 mmol) **6** in 20 mL 95% ethanol. After the solution was heated to 60 °C, the mixture was stirred at the temperature for 1 h. Then the reaction was terminated and the majority of solvent was evaporated under reduced pressure. Water was added into the reaction mixture, and the product was extracted with ethyl acetate (20 × 3 mL). The combined extracts were washed with saturated brine, dried, and evaporated under reduced pressure. The residue was subjected to chromatography using petroleum ether/ethyl acetate (5:1) as the eluent to give 50 mg of **7** as white solid. Yield: 42%, mp 285–286 °C. IR (KBr) ν: 3317, 2941, 2859, 2348, 1645, 1600, 1449, 1367, 1257, 963, 930 cm^−1^; ^1^H NMR (d_6_-DMSO, 600MHz) δ: 0.70 (3H, s, 18-CH_3_), 0.88 (3H, d, *J* = 6.6, 26- or 27-CH_3_), 0.89 (3H, d, *J* = 6.6, 26- or 27-CH_3_), 0.92 (3H, d, *J* = 6.6, 21-CH_3_), 1.08 (3H, s, 19-CH_3_), 2.07-1.99 (2H, m, ), 3.21-3.16 (1H, m, C_7a_-αH), 3.38-3.33 (1H, m, C_7a_-βH), 3.45 (1H, dd, *J* = 15.3, 4.8, C_2_-βH), 6.11 (1H, d, *J* = 1.8, C_4_-H), 6.33 (1H, s, -NH), 9.50 (H, s, =N-OH); ^13^C NMR (d_6_-DMSO, 75MHz) δ: 168.0 (6-C), 158.6 (3-C), 153.3 (5-C), 121.3 (4-C), 56.1 (17-C), 55.9 (14-C), 51.2 (9-C), 44.8 (7a-C), 42.6 (13-C), 36.0 (10-C), 35.5 (22-C), 33.9 (20-C), 30.4 (1-C), 28.2 (2-C), 27.8 (16-C), 27.8 (25-C), 23.6 (15-C), 23.1 (23-C), 22.9 (26 or 27-C), 21.8 (11-C), 20.7 (19-C), 18.9 (21-C), 12.2 (18-C); HRESI-MS (*m/z*): 429.3463 [M + H]^+^ (calcd for C_27_H_45_N_2_O_2_, 429.3481) (Supplementary Information: Figure S4).

Compounds in Scheme 2 were prepared similarly according to the procedure of Scheme 1.

#### 3.1.5. The Synthesis of 3-Acetoxy-6-aza-*B*-homocholest-7-one (**9**)

White solid, yield 93%, mp 222–223 °C; IR(KBr) ν: 3346, 2974, 2945, 2864, 1719, 1662, 1654, 1474, 1364, 1249 cm^−1^; ^1^H NMR(CDCl_3_, 300MHz) δ: 0.68 (3H, s, 18-CH_3_), 0.86 (3H, s, 19-CH_3_), 0.87 (6H, d, *J* = 6.3, 26- and 27-CH_3_), 0.90 (3H, d, *J* = 6.3, 21-CH_3_), 2.05 (3H, s, CH_3_CO-), 2.36-2.22 (2H, m, C_7a_-H), 3.47-3.40 (1H, m, C_5_-H), 4.70-4.60 (1H, m, C_3_-αH), 5.54 (1H, d, *J* = 5.1, N-H); ^13^C NMR (CDCl_3,_ 75MHz) δ: 176.2 (-CONH), 170.5 (COCH_3_), 70.9 (3-C), 58.7 (14-C), 56.7 (17-C), 56.4 (5-C), 55.6 (9-C), 42.5 (7a-C), 40.2 (13-C), 39.9 (12-C), 39.5 (24-C), 38.7 (20-C), 35.9 (10-C), 35.7 (22-C), 35.3 (1-C), 34.6 (4-C), 34.5 (8-C), 28.0 (16-C), 27.6 (25-C), 27.0 (2-C), 25.6 (23-C), 23.8 (15-C), 23.0 (11-C), 22.8 (27-C), 22.6 (C-26), 21.3 (CH_3_CO-), 18.6 (21-C), 12.4 (19-C), 11.8 (18-C); HRESI-MS (*m/z*): 460.3784 [M + H]^+^ (calcd for C_29_H_50_NO_3_, 460.3791) (Supplementary Information: Figure S5).

#### 3.1.6. The Synthesis of 3-Hydroxy-6-aza-*B*-homocholest-7-one (**10**)

White solid, yield: 76%. mp 234–235 °C. IR (KBr) ν: 3321, 2953, 2864, 1650, 1446, 1384, 1053 cm^−1^; ^1^H NMR (CDCl_3_, 600MHz) δ: 0.67 (3H, s, 18-CH_3_), 0.84 (3H, s, 19-CH_3_), 0.85 (3H, d, *J* = 6.6, 26 or 27-CH_3_), 0.86 (3H, d, *J* = 6.6, 26 or 27-CH_3_), 0.88 (3H, d, *J* = 6.6, 21-CH_3_), 2.24 (1H, t, *J* = 13.8, C_7a_-H), 2.33 (1H, d, *J* = 13.8, C_7a_-H), 3.38-3.34 (1H, m, C_5_-H), 3.61-3.57 (1H, m, C_3_-H), 5.30 (1H, s, N-H); ^13^C NMR (CDCl_3,_ 75MHz) δ: 176.3 (7-C), 68.8 (3-C), 58.8 (14-C), 56.9 (17-C), 56.5 (5-C), 55.7 (9-C), 42.5 (7a-C), 40.3 (13-C), 39.9 (12-C), 39.5 (24-C), 38.6 (4-C), 36.0 (10-C), 35.7 (22-C), 35.6 (20-C), 34.6 (1-C), 30.9 (8-C), 28.0 (2-C), 28.0 (25-C), 27.6 (16-C), 25.6 (23-C), 23.8 (15-C), 23.0 (11-C), 22.8 (27-C), 22.6 (26-C), 18.6 (21-C), 12.5 (19-C), 11.8 (18-C); LRESI-MS (*m/z*): 418.3 [M + H]^+^(Supplementary Information: Figure S6).

#### 3.1.7. The Synthesis of 6-Aza-*B*-homocholest-3,7-dione (**11**)

White solid, yield: 80%. mp 238–240 °C. IR (KBr) ν: 3346, 2966, 2864, 1723, 1658, 1458 cm^−1^; ^1^H NMR (CDCl_3_, 300MHz) δ: 0.72 (3H, s, 18-CH_3_), 0.88 (6H, d, *J* = 6.6, 26 and 27-CH_3_), 0.91 (3H, d, *J* = 6.6, 21-CH_3_), 1.07 (3H, s, 19-CH_3_), 2.52-2.22 (6H, m, C_2_-, C_4_- and C_7a_-H), 3.78-3.66 (1H, m, C_5_-H), 6.10 (1H, s, N-H); ^13^C NMR (CDCl_3_, 75MHz) δ: 207.8 (3-C), 176.4 (7-C), 58.5 (14-C), 58.0 (17-C), 56.5 (9-C), 55.7 (5-C), 43.7 (13-C ), 42.5 (7a-C), 40.2 (4-C), 39.9 (12-C), 39.5 (24-C), 39.0 (10-C), 36.8 (2-C), 36.0 (20-C), 35.9 (22-C), 35.7 (1-C), 34.7 (8-C), 28.0 (16-C), 27.6 (25-C), 25.6 (23-C), 23.8 (15-C), 23.6 (11-C), 22.8 (27-C), 22.5 (26-C), 18.6 (21-C), 12.2 (19-C), 11.8 (18-C); HRESI-MS (*m/z*): 416.3523 [M + H]^+^ (calcd for C_27_H_46_NO_2_, 416.3529) (Supplementary Information: Figure S7).

#### 3.1.8. The Synthesis of 3-Hydroximino-6-aza-*B*-homocholest-7-one (**12**)

White solid, yield: 85%, mp 248–249 °C. IR (KBr) ν: 3235, 2945, 1650, 1458, 1433, 1380, 1119, 972 cm^−1^; ^1^H NMR (CDCl_3_, 300MHz) δ: 0.70 (3H, s, 18-CH_3_), 0.84 (3H, s, 19-CH_3_), 0.85 (3H, d, *J* = 6.6, 26 or 27-CH_3_), 0.86 (3H, d, *J* = 6.6, 26 or 27-CH_3_), 0.94 (3H, d, *J* = 6.6, 21-CH_3_), 3.58-3.44 (1H, m, C_5_-H), 6.19 (1H, s, N-H); ^13^C NMR (CDCl_3_, 75MHz) δ: 176.9 (7-C), 157.5 (3-C), 58.4 (14-C), 57.4 (17-C), 56.5 (5-C), 55.7 (9-C), 42.5 (7a-C), 40.1 (13-C), 39.9 (10-C), 39.5 (12-C), 37.0 (24-C), 36.0 (22-C), 35.7 (20-C), 34.6 (8-C), 29.6 (4-C), 28.0 (16-C), 27.6 (25-C), 27.0 (2-C), 25.6 (1-C), 23.8 (23-C), 23.2 (15-C), 22.8 (26-C), 22.6 (27-C), 22.1 (11-C), 18.6 (21-C), 12.0 (19-C), 11.8 (18-C); HRESI-MS (*m/z*): 431.3630 [M + H]^+^ (calcd for C_27_H_47_N_2_O_2_, 431.3638).

#### 3.1.9. The Synthesis of 3-*O*-Methyloximino-6-aza-*B*-homocholest-7-one (**13**)

Compounds **13** and **14** were prepared similarly according to the procedure of **11**, but CH_3_ONH_2_·HCl and PhCH_2_ONH_2_·HCl were used as reagents instead of NH_2_OH·HCl.

Compound **13** was a mixture of (*E*) and (*Z*)-**13 **(ratio: *E*:*Z* = 0.9:1.1). Yield: 68%, mp 204–205 °C. IR (KBr) ν: 3425, 3222, 1954, 2869, 1673, 1048, 1016 cm^−^^1^; ^1^H NMR (CDCl_3_, 300MHz) δ: 0.66 (3H, s, 18-CH_3_), 0.83 (3H, d, *J* = 6.6, 26-CH_3_ or 27-CH_3_), 0.84 (3H, d, *J* = 6.6, 26-CH_3_ or 27-CH_3_), 0.86 (3H, d, *J* = 6.6, 21-CH_3_), 0.91 (3H, s, 19-CH_3_), 3.50-3.38 (1H, m, C_5_-H), 3.01 (*Z*: 0.55H, br d, *J* = 14.7, C_4_-H), 3.31 (*E*: 0.45H, ddd, *J* = 14.4, 5.4, 1.5, C_2_-βH), 3.786 (*Z*: 3H, s, -OCH_3_), 3.789 (*E*: 3H, s, -OCH_3_), 6.17 (*Z*: 0.55H, br s, N-H), 6.23 (0.45H, br s, N-H); ^13^C NMR (CDCl_3_, 75MHz) δ: 176.6 (7-C), 157.0 (3-C, *E*-), 156.3 (3-C, *Z*-), 61.2 (-OCH_3_), 58.6 (5-C, *Z*-), 58.4 (14-C), 57.4 (5-C, *E*-), 56.4 (17-C), 55.6 (9-C), 42.4 (7a-C), 40.2 (13-C), 40.1 (10-C), 39.9 (12-C), 39.4 (24-C), 37.2 (22-C), 36.2 (20-C), 35.9 (4-C, *Z*-), 35.7(4-C, *E*-), 34.7 (8-C, *Z*-), 34.3 (8-C, *E*-), 28.0 (16-C), 27.7 (2-C, *E*-), 27.6 (25-C), 27.1 (2-C, *Z*-), 25.6 (1-C), 23.7 (23-C), 23.1 (15-C), 22.8 (27-C), 22.6 (26-C), 20.8 (11-C), 18.6 (21-C, *E*-), 12.1 (19-C, *Z*-), 12.0 (19-C), 11.8 (18-C); HRESI-MS (*m/z*): 445.3788 [M + H]^+^ (calcd for C_28_H_49_N_2_O_2_, 445.3794) (Supplementary Information: Figure S8).

#### 3.1.10. The Synthesis of 3-*O*-Benzyloximino-6-aza-*B*-homocholest-7-one (**14**)

Compound **14** was a mixture of (*E*) and (*Z*)-**14** (ratio: *E*:*Z* = 0.9:1.1). Yield: 63%, mp 195–196 °C. IR(KBr) ν : 3424, 3224, 3088, 3031, 2950, 2868, 1672, 1452, 1366, 1351, 1048, 1015, 734, 697 cm^−1^; ^1^H NMR (CDCl_3_, 300MHz) δ: 0.68 (3H, s, 18-CH_3_), 0.858 (3H, d, *J* = 6.6, 26-CH_3_ or 27-CH_3_), 0.863 (3H, d, *J* = 6.6, 26-CH_3_ or 27-CH_3_), 0.89 (3H, d, *J* = 6.6, 21-CH_3_), 0.93 (3H, s, 19-CH_3_), 3.13 (0.5H, br d, *J* = 15.0, C_4_-H, *E*-), 3.42-3.36 (0.5H, m, C_2_-H, *Z*-), 3.52-3.42 (1H, m, C_5_-H), 5.05 (2H, s, -OCH_2_Ph), 5.90 (1H, s, N-H), 7.36-7.30 (5H, m, -C_6_H_5_); ^13^C NMR (CDCl_3,_ 75MHz): 176.4 (7-C), 157.6 (*E:* 3-C), 156.9 (*Z:* 3-C), [*Z:* 137.9, 128.4, 128.4, 128.1, 128.1, 127.8, (-C_6_H_5_)], [*E:* 137.7, 128.4, 128.4, 128.0, 128.0, 127.7, (-C_6_H_5_)], 75.5 (-OCH_2_Ph), 58.5 (*Z:* 5-C), 58.3 (14-C), 57.4 (*E:* 5-C), 56.4 (17-C), 55.7 (9-C), 42.5 (7a-C), 42.4 (13-C), 39.9 (10-C), 39.5 (12-C), 37.2 (24-C), 36.2 (22-C), 35.9 (20-C), 35.7 (8-C), 34.7 (*Z:* 4-C), 34.4 (*E:* 4-C), 28.0 (16-C), 28.0 (25-C), 27.6 (*E:* 2-C), 27.1 (*Z:* 2-C), 25.6 (1-C), 23.8 (22-C), 23.1 (15-C), 22.8 (26-C), 22.6 (27-C), 21.1 (11-C), 18.6 (21-C), 12.1 (*E:* 19-C), 12.0 (*Z:* 19-C), 11.8 (18-C); HRESI-MS (*m/z*): 521.4102 [M + H]^+^ (calcd for C_34_H_53_N_2_O_2_, 521.4107) (Supplementary Information: Figure S9).

#### 3.1.11. The Synthesis of 6-Aza-7-oxo-*B*-homocholest-3-thiosemicarbazone (**15**)

A mixture of compound **11** (100 mg, 0.24 mmol), thiosemicarbazide (24 mg, 0.26 mmol), and a few drops of glacial acetic acid (0.5 mL) in 95% ethanol (20 mL) was stirred at 60–70 °C for 10 h. After completion of the reaction, the majority of solvent was evaporated and some water was added to this solution. The mixture was extracted with CH_2_Cl_2_ and the extract was washed with saturated brine, dried with anhydrous sodium sulfate and evaporated under reduce pressure. The resulting residue was chromatographed on a column of silica gel with a mixture of DCM-methanol (20:1) to give 80 mg of compound **15**. Yield: 70%, mp 274–276 °C. Compound **15** was a mixture of (*E*)- and (*Z*)-**15 **(ratio: *E*:*Z* = 1:1). IR (KBr) ν: 3424, 3237, 2951, 2668, 1658, 1595, 1467, 1386, 1284, 1134, 1078 cm^−1^; ^1^H NMR (DMSO, 600 MHz) δ: 0.64 (3H, s, 18-CH_3_), 0.814 (3H, d, *J* = 6.6, 26 or 27-CH_3_), 0.818 (3H, d, *J* = 6.6, 26 or 27-CH_3_), 0.84 (1.5H, s, 19-CH_3_), 0.85 (1.5H, s, 19-CH_3_), 0.92 (3H, d, *J* = 6.0, 21-CH_3_), 2.69 (0.5H, d, *J* = 12.6, C_2_-H, *E*-), 2.81 (1H, br s, C_4_-H, *Z*-), 2.97 (0.5H, d, *J* = 10.8, C_4_-H, *Z*-), 3.51 (0.5H, *J* = 4.8, C_5_-H, *E*-), 3.57 (0.5H, *J* = 4.8, C_5_-H, *Z*-), 7.13 (1H, br s, -NH_2_), 7.29 (0.5H, br s, CON-H-), 7.36 (1H, br s, -NH_2_), 7.43 (0.5H, br s, CON-H-), 9.57 (0.5H, br s, -NH-, *E*-), 10.20 (0.5H, br s, -NH-, *Z*-); ^13^C NMR (DMSO, 150 MHz) δ: 178.5 (C=S), 177.4 (7-C, *Z*-), 177.1 (7-C, *E*-), 156.3 (3-C, *Z*-), 155.1 (3-C, *E*-), 59.0 (5-C, *Z*-), 58.3 (5-C, *E*-), 58.2 (14-C), 56.5 (17-C), 55.6 (9-C), 42.6 (7a-C), 40.3 (10-C, *Z*-), 40.2 (10-C, *E*-), 39.9 (13-C), 39.6 (12-C), 39.4 (24-C), 37.7 (4-C, *Z*-), 37.2 (4-C, *E*-), 36.2 (2-C, *E*-), 36.0 (22-C), 35.8 (20-C), 34.7 (8-C), 30.6 (2-C, *Z*-), 28.1 (16-C), 27.7 (1-C), 25.8 (25-C), 23.9 (23-C), 23.4 (15-C), 23.3 (11-C), 22.7 (27-C), 22.9 (26-C), 18.7 (21-C), 12.6 (19-C, *Z*-), 12.4 (19-C, *E*-), 12.0 (18-C); HRESI-MS (*m/z*): 489.3620 [M + H]^+^ (calcd for C_28_H_49_N_4_OS, 489.3627) (Supplementary Information: Figure S10).

Compounds in Scheme 3 were prepared similarly according to the procedure of Scheme 1.

#### 3.1.12. 3-Acetoxy-7a-aza-*B*-homocholest-5-ene-7-one (**17**)

Yellow solid, yield: 72%, mp192–194 °C. IR (KBr) ν: 3424, 2942, 2868, 1740, 1659, 1617, 1442 cm^−1^; ^1^H NMR (300 MHz, CDCl_3_) δ: 0.69 (3H, s, 18-CH_3_), 0.869 (3H, d, *J* = 6.6, 26 or 27-CH_3_), 0.874 (3H, s, *J* = 6.6, 26 or 27-CH_3_), 0.92 (3H, d, *J* = 6.6, 21-CH_3_), 1.27 (3H, s, 19-CH_3_), 2.05 (3H, s, 2′-CH_3_), 2.46 (1H, dd, *J* = 13.2, 3.6, C_4_-βH), 2.61 (1H, t, *J* = 11.7, C_4_-αH), 3.29 (1H, t, *J* = 8.4, C_8_-H), 4.76-4.65 (1H, m, C_3_-αH), 5.66 (1H, br s, -NH-), 5.83 (1H, s, C_6_-H); ^13^C NMR (75 MHz, CDCl_3_) δ: 170.3 (1′-C), 167.4 (7-C), 156.1 (5-C), 122.5 (6-C), 72.5 (3-C), 55.7 (14-C), 55.4 (17-C), 51.7 (8-C), 48.9 (9-C), 43.8 (24-C), 41.8 (12-C), 41.6 (13-C), 39.4 (15-C), 38.6 (10-C), 35.9 (4-C), 35.5 (1-C), 35.4 (22-C), 28.0 (20-C), 27.8 (25-C), 27.0 (2-C), 25.1 (16-C), 23.8 (23-C), 23.2 (11-C), 22.8 (26-C), 22.5 (27-C), 21.3 (2′-C), 19.3 (21-C), 18.6 (19-C), 11.4 (18-C); HRESI-MS (*m/z*): 458.3623 [M + H]^+^ (calcd for C_29_H_48_NO_3_, 458.3634) (Supplementary Information: Figure S11).

#### 3.1.13. 3-Hydroxy-7a-aza-*B*-homocholest-5-ene-7-one (**18**)

Yellow solid, yield: 74.5%, mp 207–209 °C; IR (KBr) ν: 3383, 2946, 2860, 1663, 1601, 1438 cm^−1^; ^1^H NMR (300 MHz, CDCl_3_) δ: 0.65 (3H, s, 18-CH_3_), 0.837 (3H, d, *J* = 6.6, 26 or 27-CH_3_), 0.842 (3H, s, *J* = 6.6, 26 or 27-CH_3_), 0.89 (3H, d, *J* = 6.6, 21-CH_3_), 1.24 (3H, s, 19-CH_3_), 2.40 (1H, dd, *J* = 13.2, 3.3, C_4_-βH), 2.53 (1H, t, *J* = 11.7, 3.6, C_4_-αH), 3.25 (1H, t, *J* = 8.1, C_8_-H), 3.65-3.54 (1H, m, C_3_-αH), 5.77 (1H, s, C_6_-H), 6.01 (1H, br s, -NH-); ^13^C NMR (75 MHz, CDCl_3_) δ: 168.2 (7-C), 158.6 (5-C), 121.0 (6-C), 70.6 (3-C), 55.6 (14-C), 55.1 (17-C), 51.6 (8-C), 49.1 (9-C), 46.3 (13-C), 44.2 (4-C), 41.7 (24-C), 39.4 (12-C), 38.5 (15-C), 36.0 (1-C), 35.9 (22-C), 35.5 (20-C), 30.5 (10-C), 28.0 (2-C), 27.8 (25-C), 25.1 (16-C), 23.8 (23-C), 23.1 (11-C), 22.8 (26-C), 22.5 (27-C), 19.4 (21-C), 18.6 (19-C), 11.3 (18-C); HRESI-MS (*m/z*): 416.3515 [M + H]^+^ (calcd for C_27_H_46_NO_2_, 416.3529) (Supplementary Information: Figure S12).

#### 3.1.14. 7a-Aza-*B*-homocholest-4-ene-3,7-dione (**19**)

Faint yellow solid, yield: 43.0%, mp 203–205 °C; IR (KBr) ν: 3367, 2958, 2860, 1675, 1462 cm^−1^; ^1^H NMR (300 MHz, CDCl_3_) δ: 0.72 (3H, s, 18-CH_3_), 0.845 (3H, d, *J* = 6.6, 26 or 27-CH_3_), 0.850 (3H, s, *J* = 6.6, 26 or 27-CH_3_), 0.90 (3H, d, *J* = 6.6, 21-CH_3_), 1.27 (3H, s, 19-CH_3_), 2.37-2.28 (2H, m, C_2_-H), 2.94 (1H, d, *J* = 12.6, C_6_-βH), 3.55 (1H, d, *J* = 12.6, C_6_-αH), 3.52-3.46 (1H, m, C_8_-H), 5.82 (1H, br s, -CONH-), 5.93 (1H, s, C_4_-H); ^13^C NMR (75 MHz, CDCl_3_) δ: 198.4 (3-C), 172.5 (7-C), 162.8 (5-C), 126.9 (4-C), 55.8 (17-C), 54.0 (14-C), 52.4 (8-C), 49.2 (9-C), 42.7 (6-C), 41.6 (13-C), 41.3 (24-C), 39.4 (12-C), 38.2 (10-C), 35.9 (15-C), 35.5 (1-C), 34.1 (22-C), 33.4 (20-C), 28.0 (2-C), 27.9 (25-C), 27.7 (16-C), 24.8 (23-C), 23.8 (11-C), 22.8 (26-C), 22.6 (27-C), 21.2 (21-C), 18.6 (19-C), 11.7 (18-C); HRESI-MS (*m/z*): 414.3362 [M + H]^+^ (calcd for C_28_H_47_N_4_OS, 414.3372) (Supplementary Information: Figure S13).

#### 3.1.15. 3-Hydroximino-7a-aza-*B*-homocholest-4-ene-7-one (**20**)

Faint yellow solid, yield: 77.3%, mp 204–206 °C. IR (KBr) ν: 3268, 2949, 2868, 1658, 1657, 1462, 1366 cm^−1^; ^1^H NMR (CDCl_3_, 300MHz) δ: 0.73 (3H, s, 18-CH_3_), 0.88 (6H, d, *J* = 6.6, 26- and 27-CH_3_), 0.93 (3H, d, *J* = 6.6, 21-CH_3_), 1.22 (3H, s, 19-CH_3_), 2.33-2.22 (2H, m, C_2_-H), 2.79 (1H, br d, *J* = 17.4, C_2_-H), 2.94 (1H, d, *J* = 13.5, C_6_-βH), 3.47 (1H, d, *J* = 13.5, C_6_-αH), 3.52-3.45 (1H, m, C_8_-H), 5.77 (1H, br s, -CONH-), 6.12 (1H, s, C_4_-H), 6.84 (1H, s, -OH); ^13^C NMR (CDCl_3_, 75MHz) δ: 174.3 (7-C), 156.1 (3-C), 147.5 (5-C), 121.8 (4-C), 55.9 (14-C), 54.4 (17-C), 52.5 (8-C), 50.0 (13-C), 49.1 (9-C), 42.8 (6-C), 41.0 (24-C), 40.6 (10-C), 39.4 (12-C), 38.5 (15-C), 35.9 (22-C), 35.5 (20-C), 33.0 (1-C), 29.7 (25-C), 28.0 (2-C), 27.7 (16-C), 24.9 (23-C), 23.8 (11-C), 22.8 (26-C), 22.5 (27-C), 21.0 (19-C), 18.6 (21-C), 11.7 (18-C); HRESI-MS (*m/z*): 429.3463 [M + H]^+^ (calcd for C_27_H_45_N_2_O_2_, 429.3481).

#### 3.1.16. 7-Oxo-7a-aza-*B*-homocholest-4-ene-3-thiosemicarbazone (**21**)

Faint yellow solid, yield: 31%, mp 230-231 °C; IR(KBr) ν: 3436, 2954, 2856, 1659, 1573, 1409 cm^−1^; ^1^ H NMR (CDCl_3_, 300MHz) δ: 0.75 (3H, s, 18-CH_3_), 0.88 (3H, d, *J* = 6.6, 26 or 27-CH_3_), 0.89 (3H, d, *J* = 6.6, 26 or 27-CH_3_), 0.94 (3H, d, *J* = 6.3, 21-CH_3_), 1.26 (3H, s, 19-CH_3_), 2.97 (1H, d, *J* = 13.2, C_6_-βH), 3.51 (1H, d, *J* = 13.2, C_6_-αH), 3.56-3.48 (1H, m, C_8_-H), 5.28 (1H, br s, -CONH-), 6.10 (1H, s, C_4_-H), 6.37 (1H, br s, -NH_2_), 7.25 (1H, br s, -NH_2_), 8.64 (1H, s, -NH-CS); ^13^C NMR (CDCl_3_, 75MHz) δ: 178.7 (C=S), 173.6 (7-C), 150.5 (5-C), 148.9 (3-C), 124.2 (4-C), 55.9 (14-C), 54.3 (17-C), 52.5 (8-C), 49.2 (9-C), 42.8 (6-C), 41.1 (13-C), 40.5 (24-C), 39.4 (12-C), 38.4 (15-C), 35.9 (22-C), 35.5 (20-C), 32.8 (10-C), 29.7 (1-C), 28.0 (25-C), 24.9 (16-C), 23.8 (23-C), 22.8 (26-C), 22.5 (27-C), 22.4 (2-C), 21.0 (11-C), 20.0 (21-C), 18.6 (19-C), 11.7 (18-C); HRESI-MS (*m/z*): 487.3491 [M + H]^+^ (calcd for C_28_H_47_N_4_OS, 487.3471) (Supplementary Information: Figure S14).

### 3.2. Biology

#### 3.2.1. Assay for Cell Viability

The cell proliferation assay was undertaken by a MTT method using 96-well plates on a MLLTISKAN MK3 analysis spectrometer. GNE 2 (nasopharyngeal carcinoma), SPC-A (lung carcinoma) and Tu 686 (laryngic carcinoma) cell lines were obtained by Guangxi Medical University (Guangxi, China); MGC 7901 (human gastric carcinoma) and HeLa (human cervical carcinoma) cell lines were obtained by Guangxi Traditional Chinese Medical University. Cells were grown in RPMI-1640 supplemented with 10% cosmic calf serum (Hyclone) and antibiotics in a humidified atmosphere of 5% CO_2_ at 37 °C. The viability of these cells was determined using the colorimetric CellTiter 96 aqueous Cell Proliferation Assay (MTT) according to the instruction provided by the manufacturer (Promega, Madison, WI, USA). Briefly, cells (1–3 × 10^4^ cells per well) were seeded in 96 wells plates. One day after seeding, the cells were treated with or without different concentration of each compound and reincubated for 72 h. After the cells were washed with sterile phosphate buffer saline (PBS), 190 µL of RPMI-1640 and 10 µL of the tetrazolium dye (MTT) (5 mg/mL) solution were added to each well, and the cells were incubated for an additional 4 h. The medium was discarded; 200 µL of DMSO was added to dissolve the purple formazan crystals formed. The absorbance (A) at 492 nm was measured using a MLLTISKAN MK3 analysis spectrometer. The IC_50_ value was calculated in µmol/L in Table 1 as the concentration of drug yielding 50% cell survival. The effect of compound **5** on the morphology of treated human carcinoma cells was investigated by the light microscope and then photographed by TE2000-U Nikon (Tokyo, Honshu, Japan) inverted microscope.

#### 3.2.2. 3D Multicellular Spheroids

H292 (lung adenocarcinoma) cancer cells were obtained from ATCC, Manassas, VA. C. Monolayer cultures were incubated in RPMI-1640 supplemented with 10% fetal bovine serum, 100 units/mL penicillin. The cell cultures were kept at 37 °C in a humidified 5% CO_2_ and 95% air incubator. Uniform single-spheroid H292 lung carcinoma cells were cultured as follows. The 96-well flat-bottom plates were coated with 70 μL of a 1.5% agarose (weight/volume) solution in distilled water (freshly autoclaved). During the coating process, the agarose solution was maintained at ≥60 °C followed by cooling and setting at room temperature for 40 min. Then the cells were plated at a density of 5000 cells/well in 80 μL of RPMI-1640 (10% FBS), and allowed to form spheroid in 48 h. The spheroids were then treated with 20 μL of a 25% solution of growth factor–reduced Matrigel^™^ in cell culture medium, resulting in a final volume of 100 μL with 5% Matrigel. Spheroids were cultured for one more day to reach an average diameter of 100 μm under standard tissue culture conditions (37 °C, 5% CO2). For drug treatment, 100 μL fresh medium with various concentration of drugs were added at day 3 (final matrigel concentration became 2.5% as well). Spheroid morphological images in 96-well microplate were carried out manually on an inverted microscope equipped with camera. Spheroid diameters and volumes were determined from their images. The treatment was quart replicated, and the spheroid images were taken every other day. The suppression of the spheroid growth was normalized with control treatment (0.1% DMSO) [21,22]. 

#### 3.2.3. Annexin V Staining Assay

Apoptosis was detected with an annexin V-FITC kit purchased from BD Pharmingen (San Diego, CA, USA) according to the manufacturer’s instructions. SGC-7901 cells were seeded in 35 mm culture dishes and allowed to attach overnight. The cells were treated with different concentration of compound **5** for 24 h respectively, collected, and washed twice with PBS. To detect early and late apoptosis, both adherent and floating cells were harvested together and resuspended in annexin V binding buffer at a concentration of 10^6^ cells/mL. Subsequently, 5 μL of FITC-conjugated annexin V and 5 μL of propidium iodide were added to 100 μL of the cell suspension (10^5^ cells). The cells were incubated for 15 min at room temperature in the dark. Finally, 400 μL of annexin V binding buffer was added to each tube, and cells were analyzed by a two color cytometry using FACS Calibur (Becton Dickinson, Biosciences, Franklin Lakes, NJ, USA).

## 4. Conclusions

We have prepared a series of aza-*B*-homocholestane derivatives having different substituted groups at 3-postion of the steroidal nucleus, taking analogues of marine steroidal oximes as precursors. The antiproliferative activity of the synthesized compounds against SGC-7901, HeLa, Bel-7404, GNE 2, SPC-A and Tu 686 cell lines was investigated. The results showed that aza-*B*-homocholestane derivatives possessing 3-hydroxyl, 3-hydroximino and 3-thiosemicarbazone groups showed remarkable cytotoxic activity. In the synthesized compounds, compounds **5**, **10**, **12**, **15** and **18 **were found to be the most potent compounds as anticancer agents, and they displayed a similar antiproliferative activity as cisplatin did. The result of 3D multicellular spheroids screening of **15** showed also distinct antiproliferative activity, and Annexin V staining assay indicated that compound **5 **was able to effectively induce tumor cells apoptosis. Compounds **5** and **15** are now submitted to further acute toxicity and antitumor activity studies in animal models, and the relative possible results will be reported in due course. Our findings provide new evidence showing the relationship between the chemical structure and biological activities, and may be useful for the design of novel chemotherapeutic drugs. 

## Conflicts of Interest

The authors declare no conflict of interest.

## Supplementary Files

Supplementary File 1 Supplementary Information (PDF, 2043 KB)
