# Supplementary material for: Synthesis and Evaluation of Some New Aza-B-homocholestane Derivatives as Anticancer Agents
Source: Mar Drugs. 2014 Mar 25;12(4):1715–31. doi: 10.3390/md12041715 (PMC4012452; doi:10.3390/md12041715)
Supplement: Supplementary File 1 — Supplementary Information (PDF, 2043 KB) [file marinedrugs-12-01715-s001.pdf]

Supplementary Information

Figure S1. The NMR spectrum of 3-acetoxy-7-aza-*B*-homocholest-4-en-6-one (**4**).
Figure S2. The NMR spectrum of 3-hydroxy-7-aza-*B*-homocholest-4-en-6-one (**5**).
Figure S3. The NMR spectrum of 7-aza-*B*-homocholest-4-en-3,6-dione (**6**).
Figure S4. The NMR spectrum of 3-hydroximino-7-aza-*B*-homocholest-4-en-6-one (**7**).
Figure S5. The NMR spectrum of 3-acetoxy-6-aza-*B*-homocholest-7-one (**9**).
Figure S6. The NMR spectrum of 3-hydroxy-6-aza-*B*-homocholest-7-one (**10**).
Figure S7. The NMR spectrum of 6-aza-*B*-homocholest-3,7-dione (**11**).
Figure S8. The NMR spectrum of 3-*O*-methyloximino-6-aza-*B*-homocholest-7-one (**13**).
Figure S9. The NMR spectrum of 3-*O*-benzyloximino-6-aza-*B*-homocholest-7-one (**14**).
Figure S10. The NMR spectrum of 6-aza-7-oxo-*B*-homocholest-3-thiosemicarbazone (**15**).
Figure S11. The NMR spectrum of 3-acetoxy-7a-aza-*B*-homocholest-5-en-7-one (**17**).
Figure S12. The NMR spectrum of 3-hydroxy-7a-aza-*B*-homocholest-5-en-7-one (**18**).
Figure S13. The NMR spectrum of 7a-aza-*B*-homocholest-4-en-3,7-dione (**19**).
Figure S14. The NMR spectrum of 7-oxo-7a-aza-*B*-homocholest-4-en-3-thiosemicarbazone (**21**).

Figure S1. The NMR spectrum of 3-acetoxy-7-aza-*B*-homocholest-4-en-6-one (**4**).

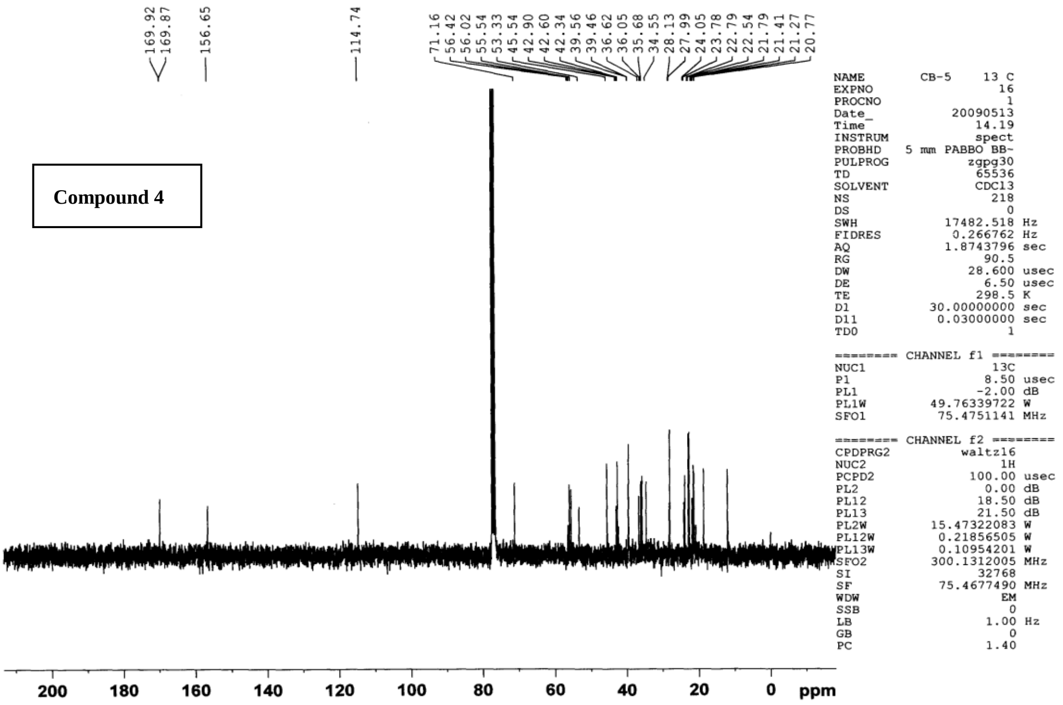

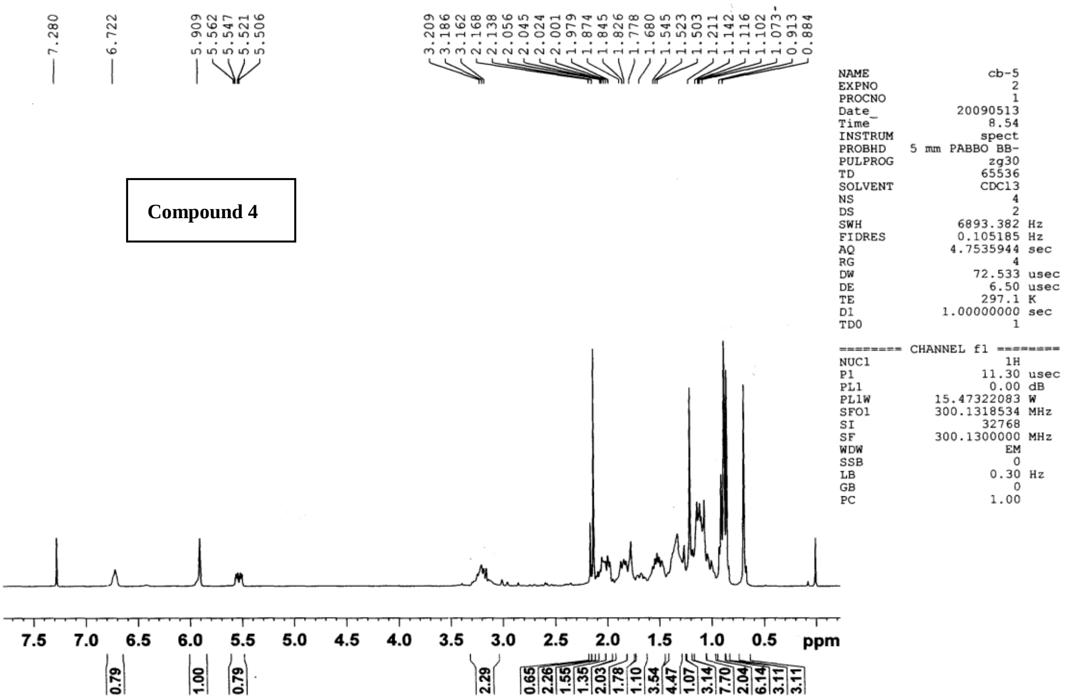

Figure S2. The NMR spectrum of 3-hydroxy-7-aza-*B*-homocholest-4-en-6-one (**5**).

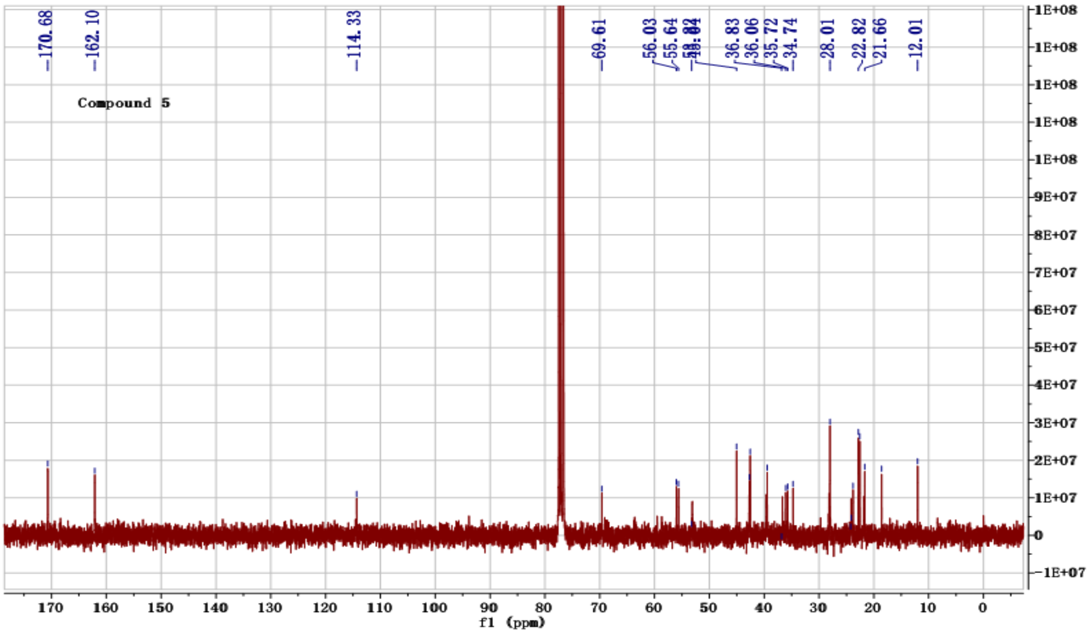

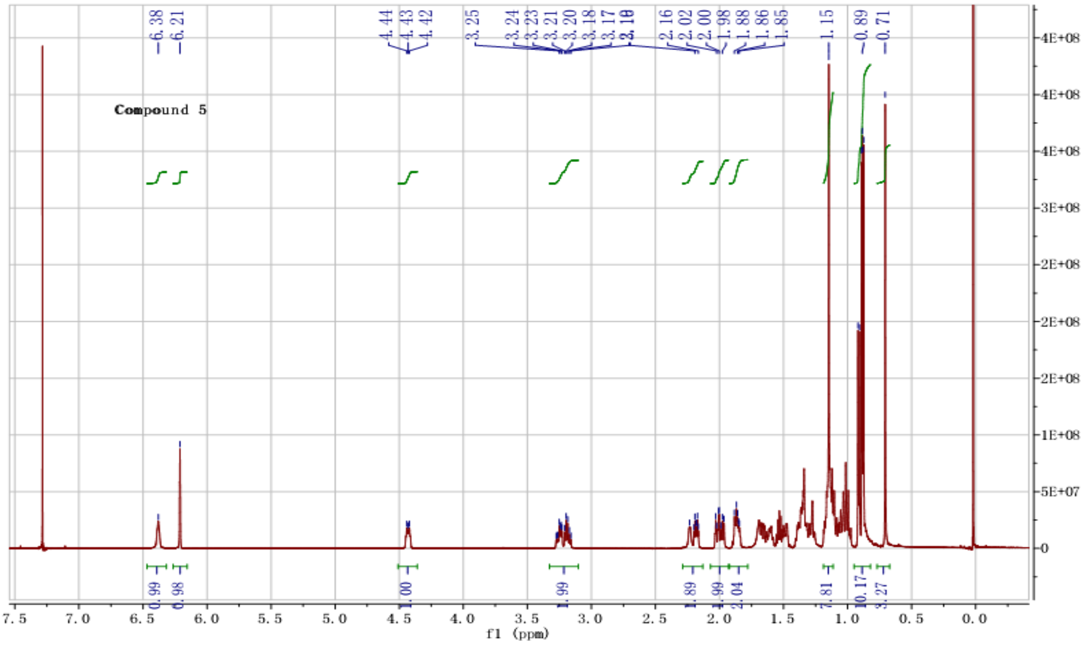

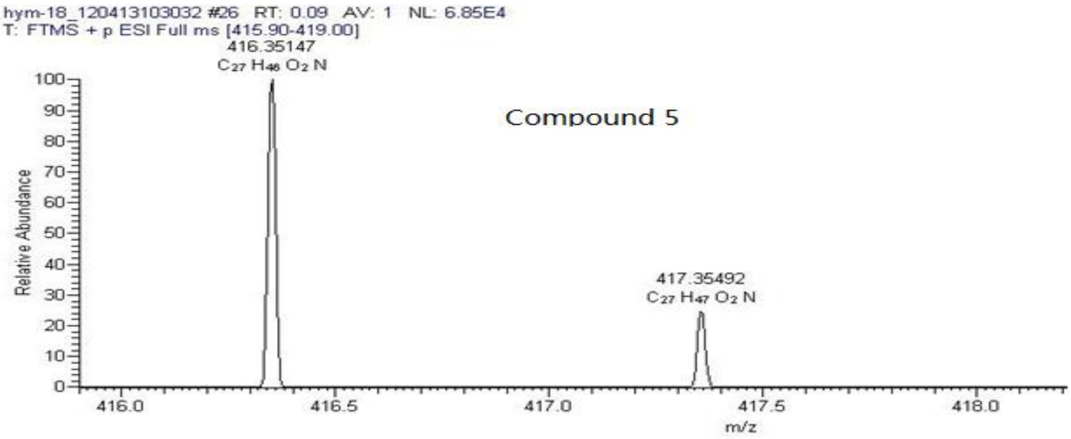

Figure S3. The NMR spectrum of 7-aza-*B*-homocholest-4-en-3,6-dione (**6**).

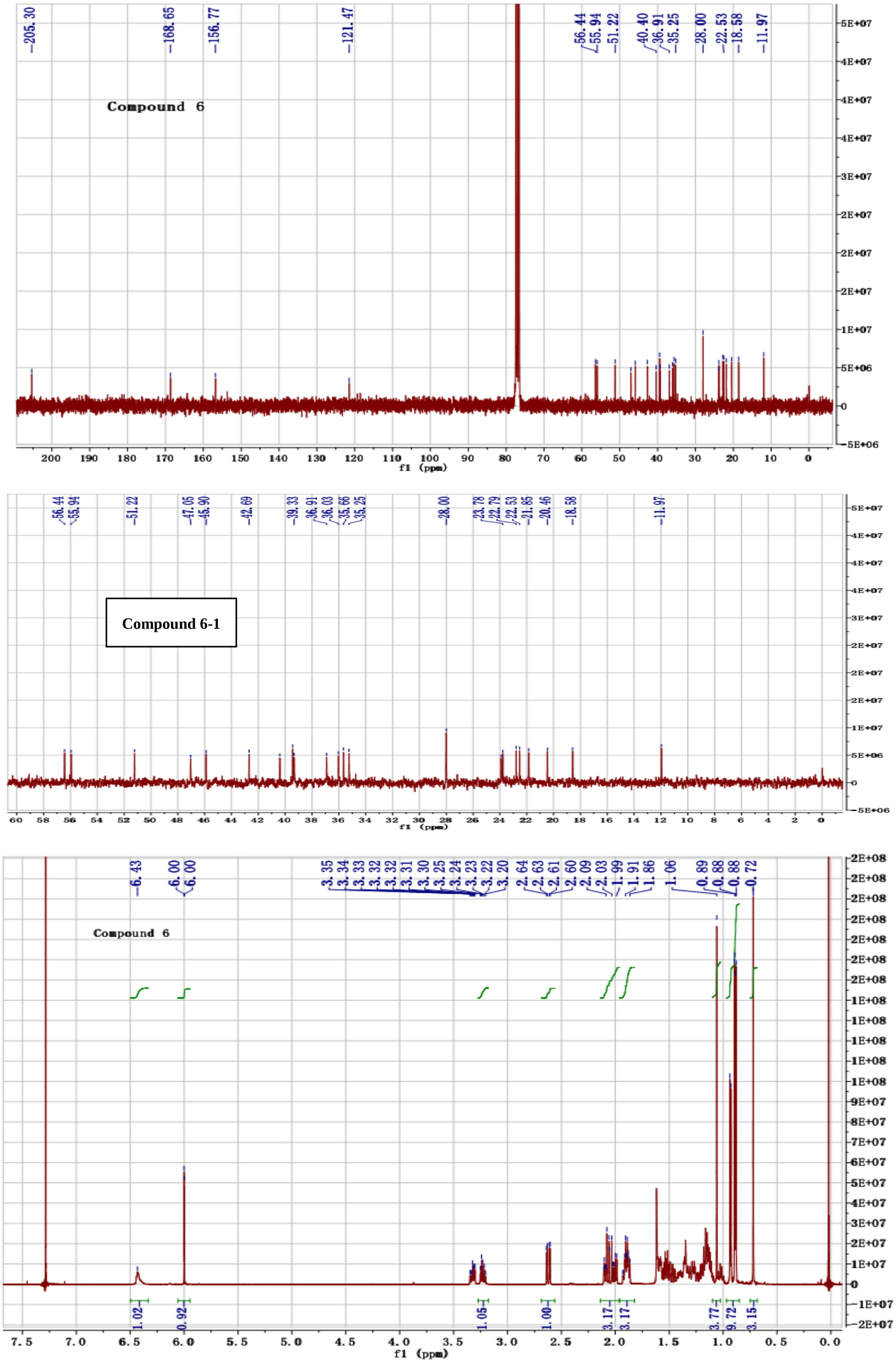

Figure S4. The NMR spectrum of 3-hydroximino-7-aza-*B*-homocholest-4-en-6-one (**7**).

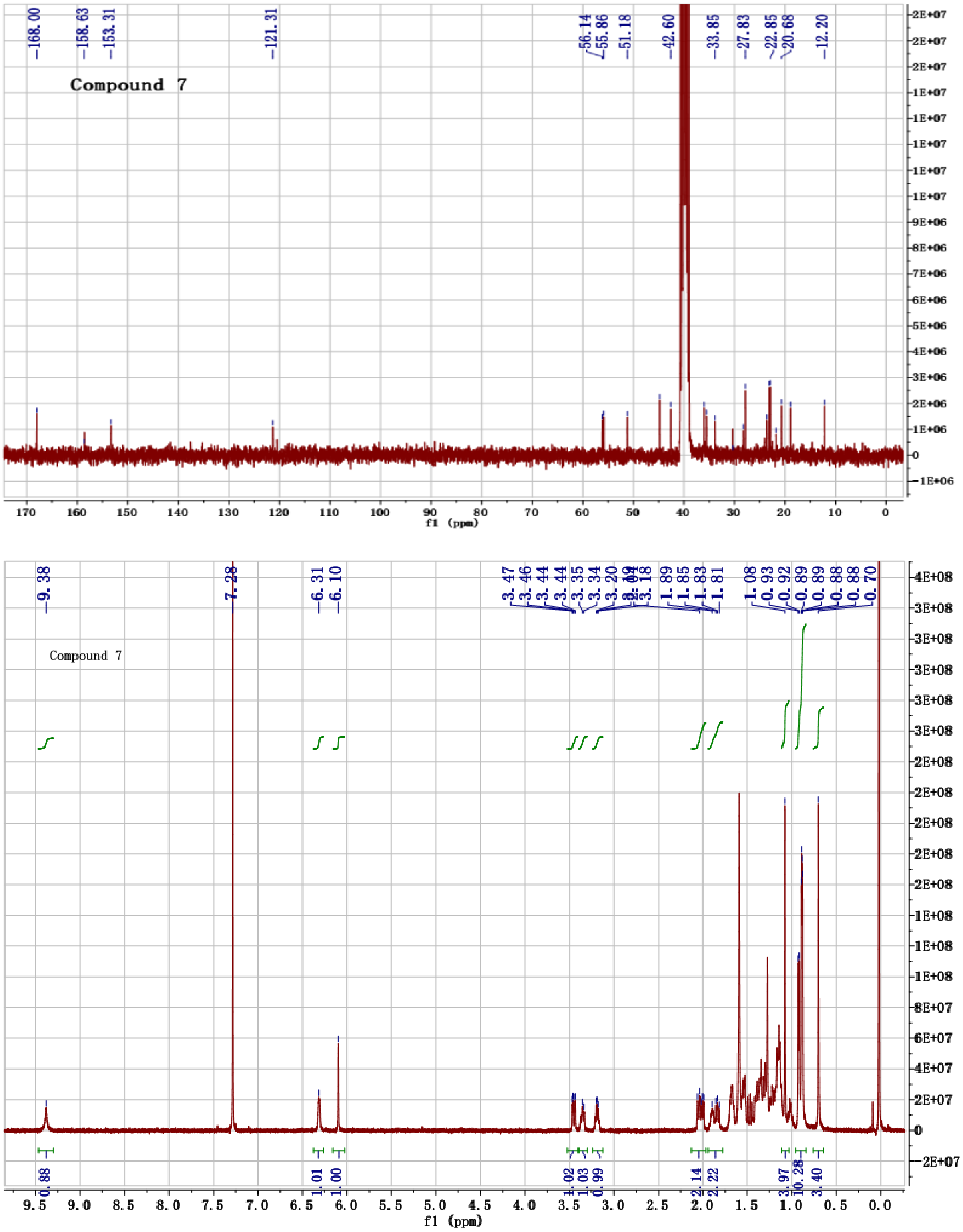

Figure S5. The NMR spectrum of 3-acetoxy-6-aza-*B*-homocholest-7-one (**9**).

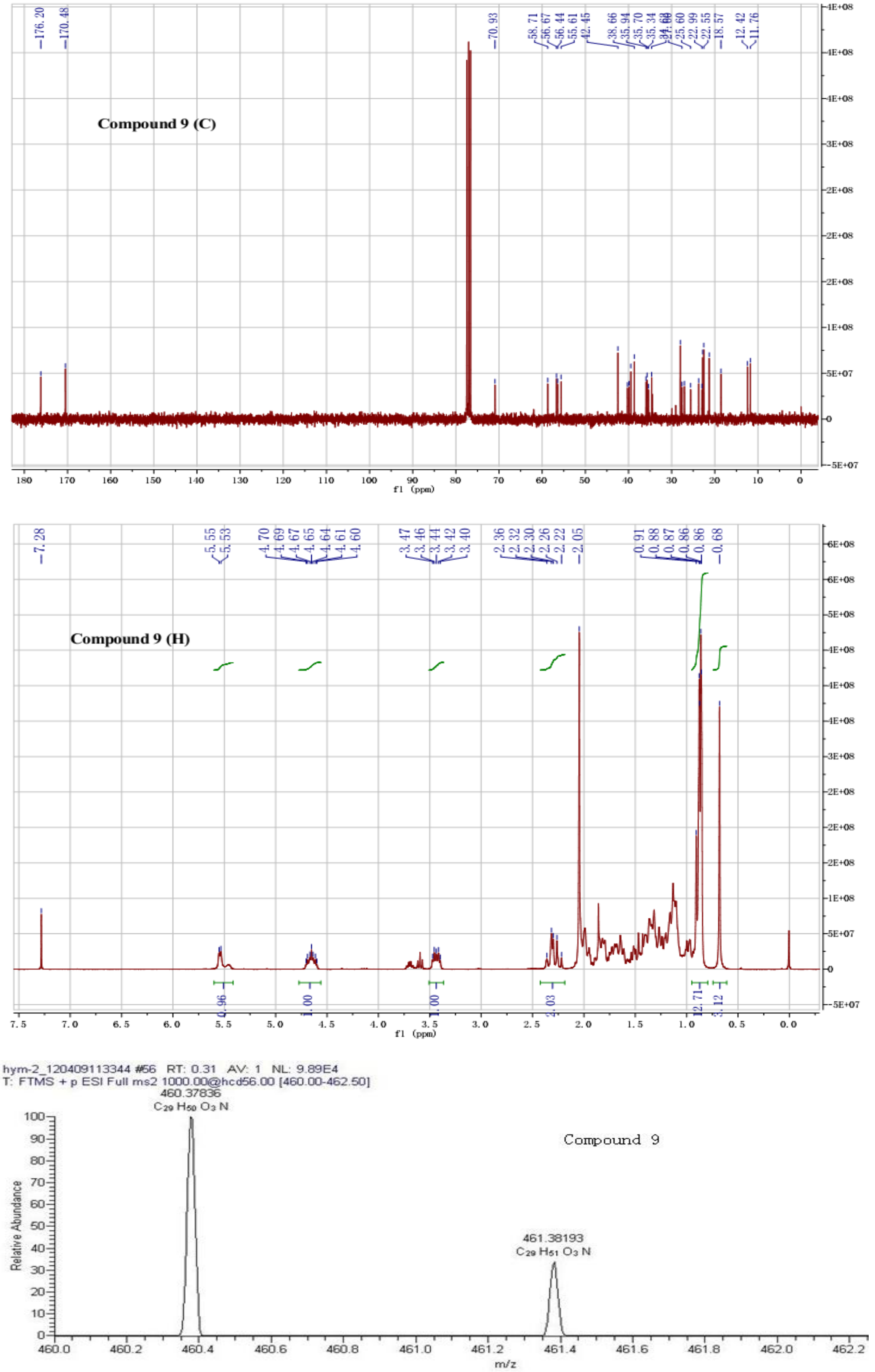

Figure S6. The NMR spectrum of 3-hydroxy-6-aza-*B*-homocholest-7-one (**10**).

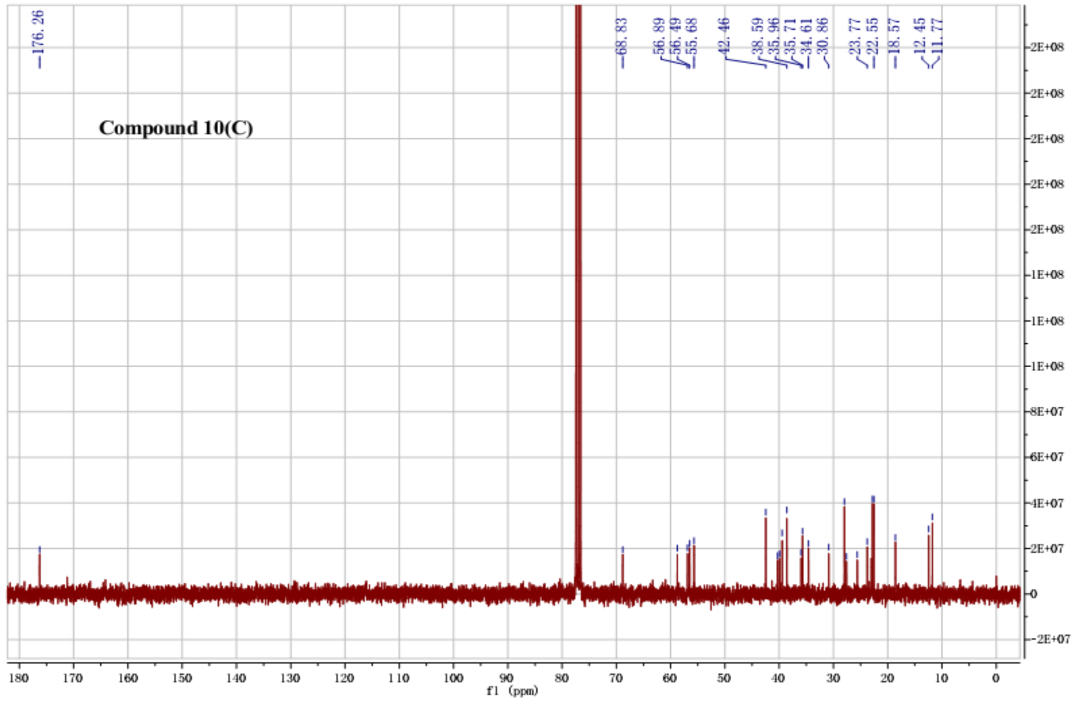

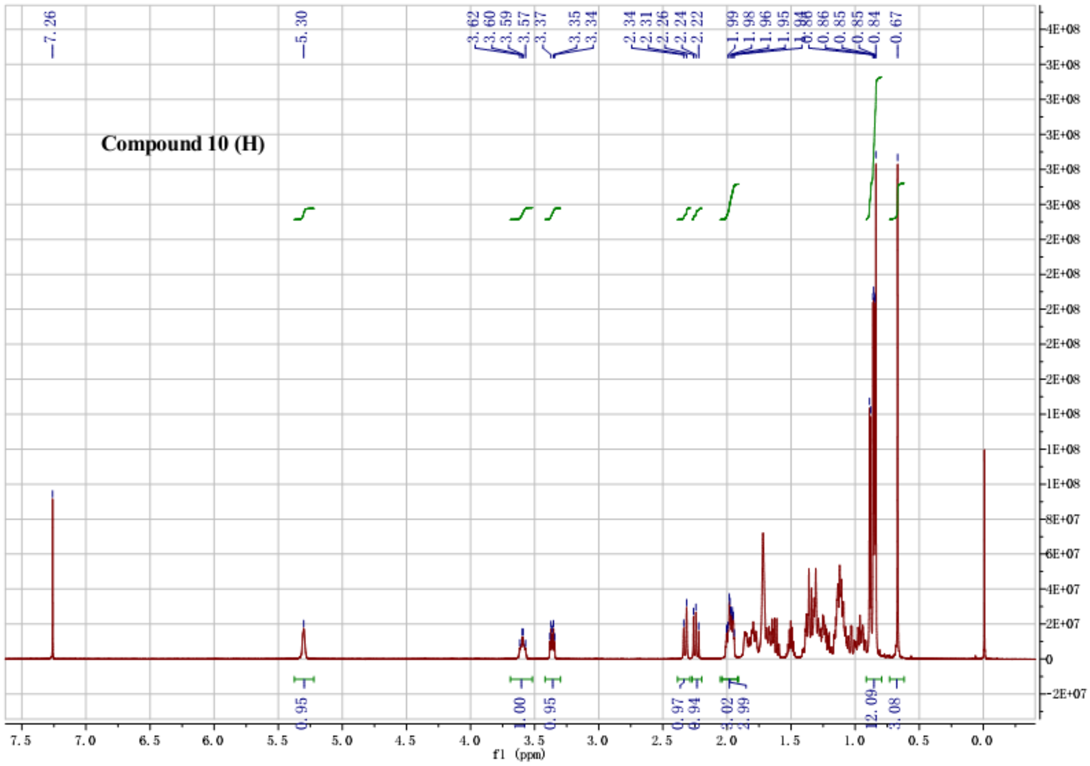

Figure S7. The NMR spectrum of 6-aza-*B*-homocholest-3,7-dione (**11**).

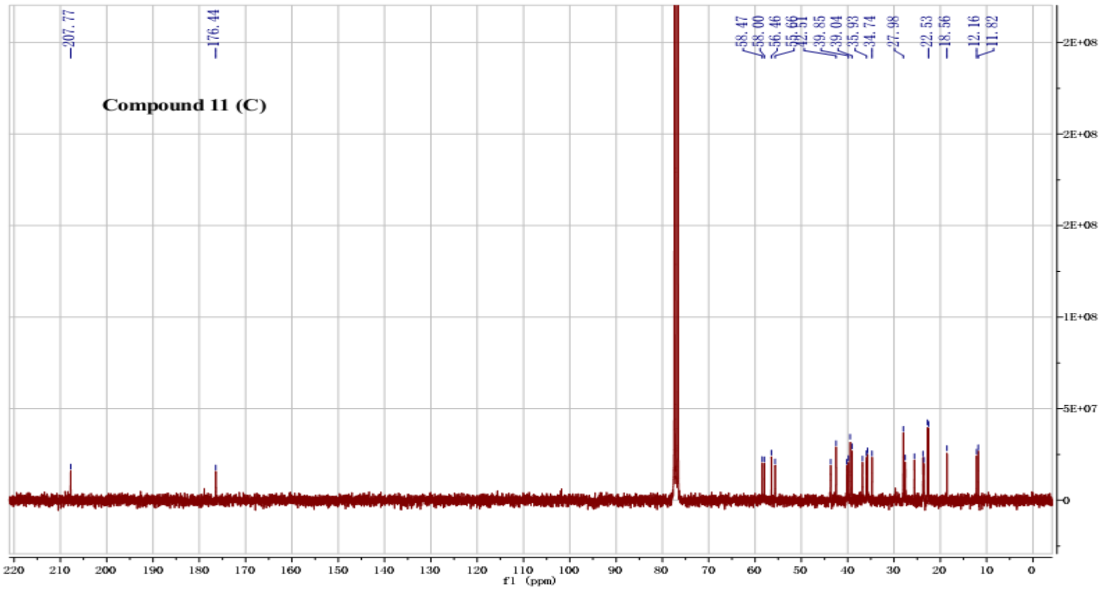

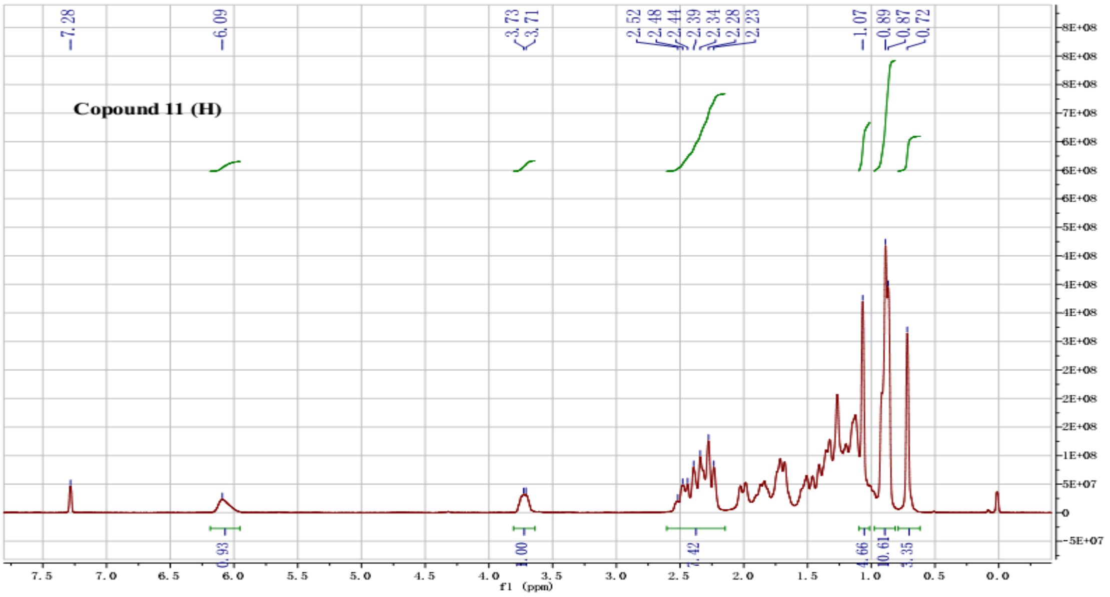

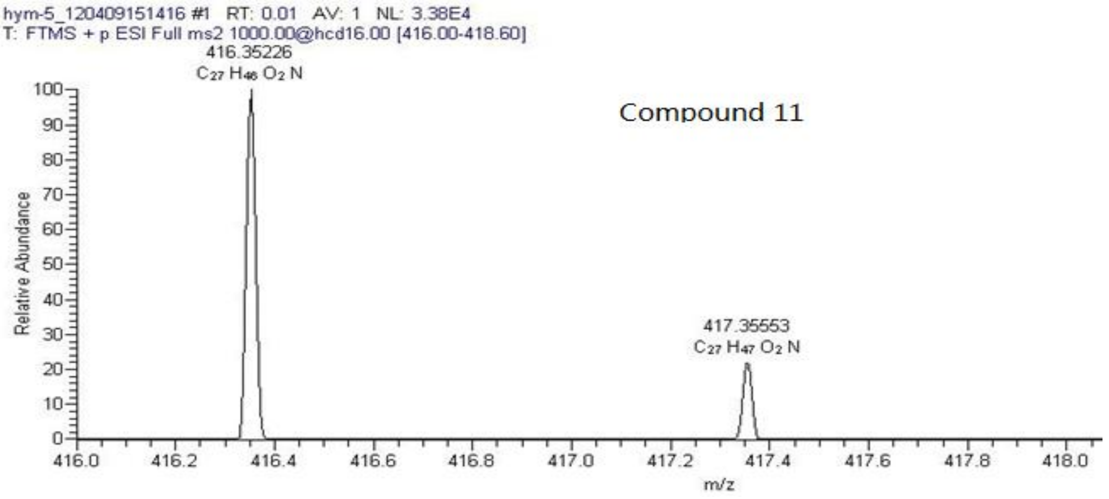

Figure S8. The NMR spectrum of 3-*O*-methyloximino-6-aza-*B*-homocholest-7-one (**13**).

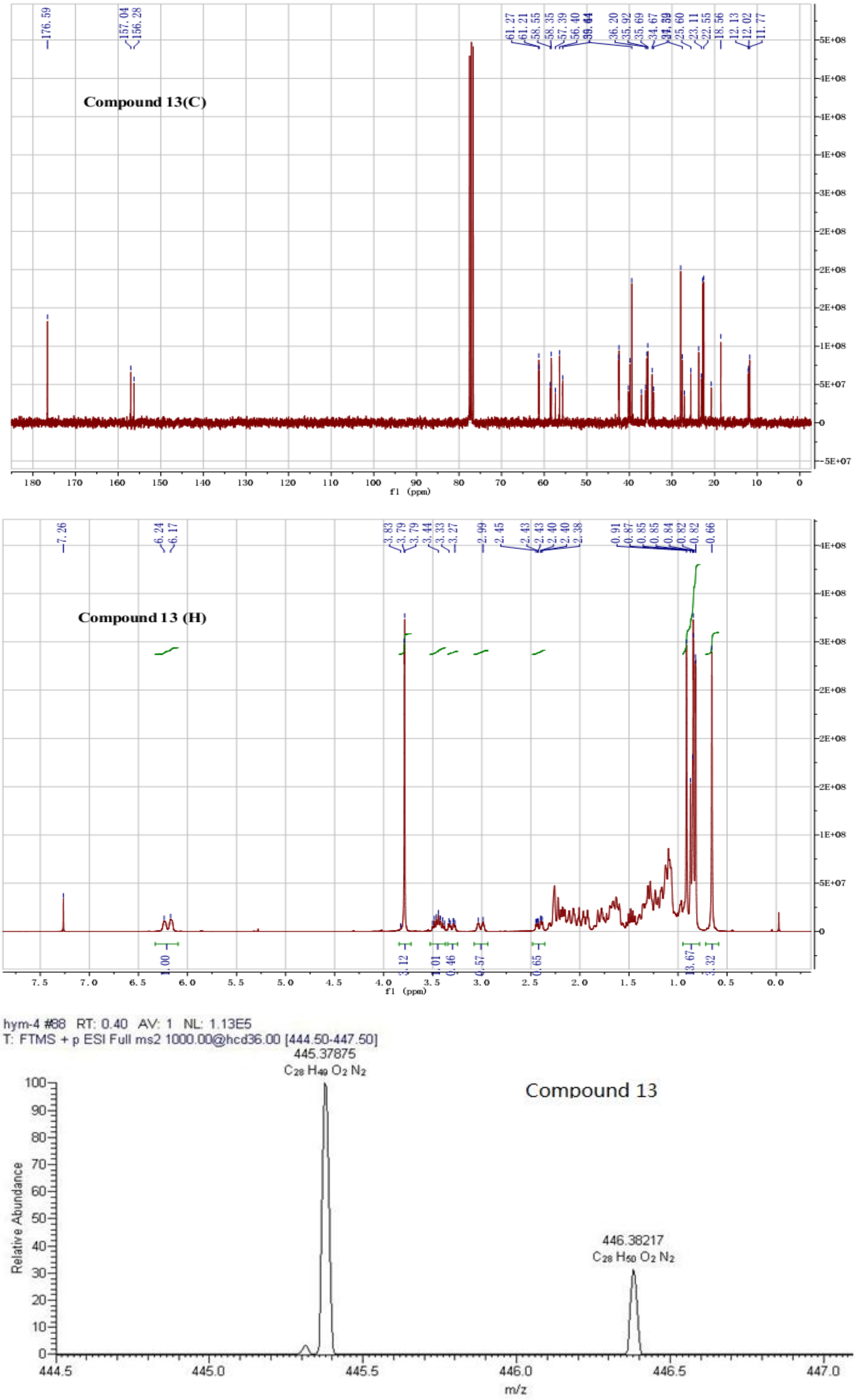

Figure S9. The NMR spectrum of 3-*O*-benzyloximino-6-aza-*B*-homocholest-7-one (**14**).

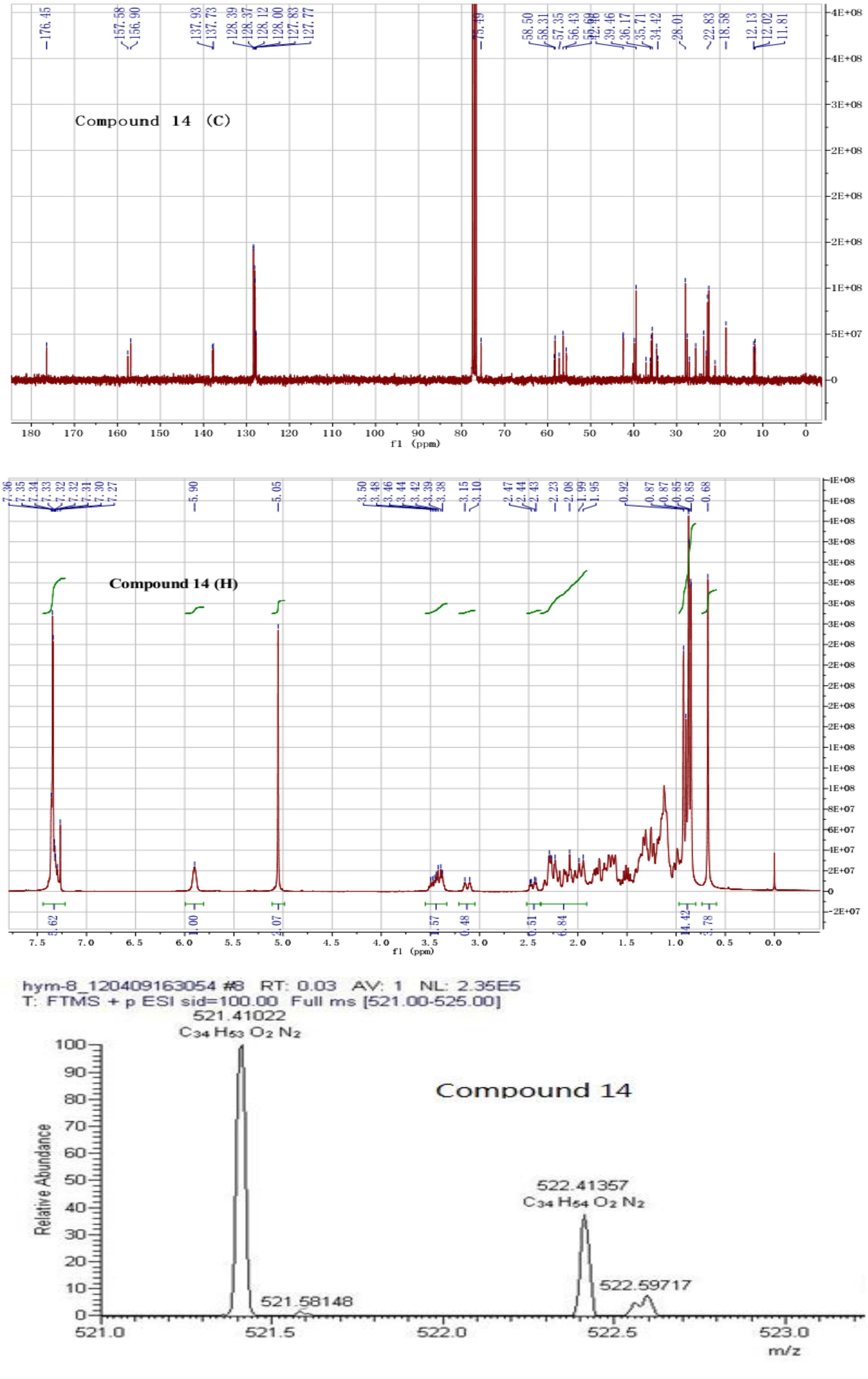

Figure S10. The NMR spectrum of 6-aza-7-oxo-*B*-homocholest-3-thiosemicarbazone (**15**).

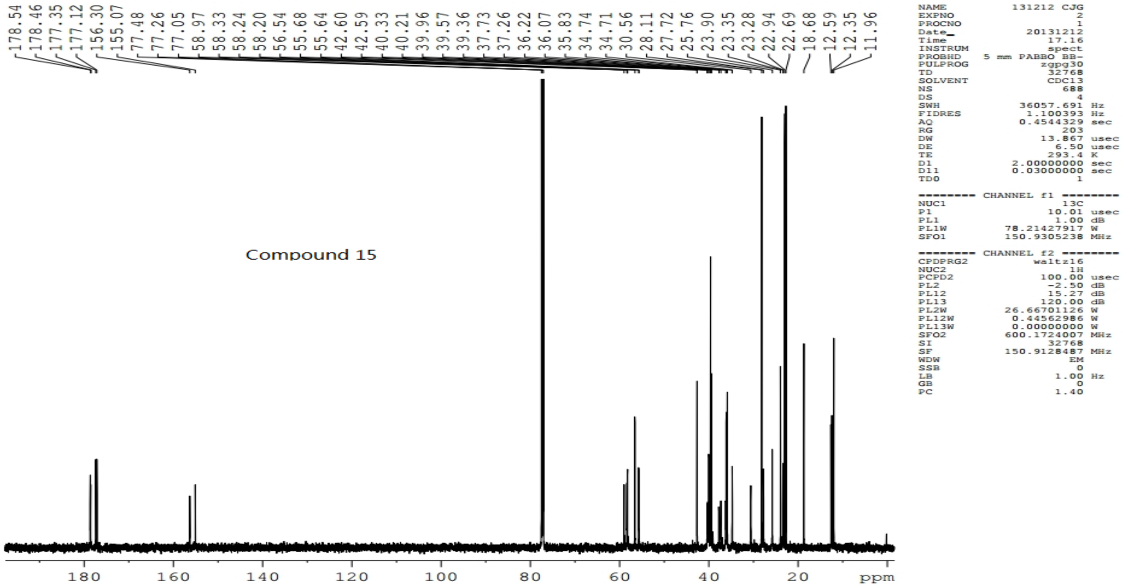

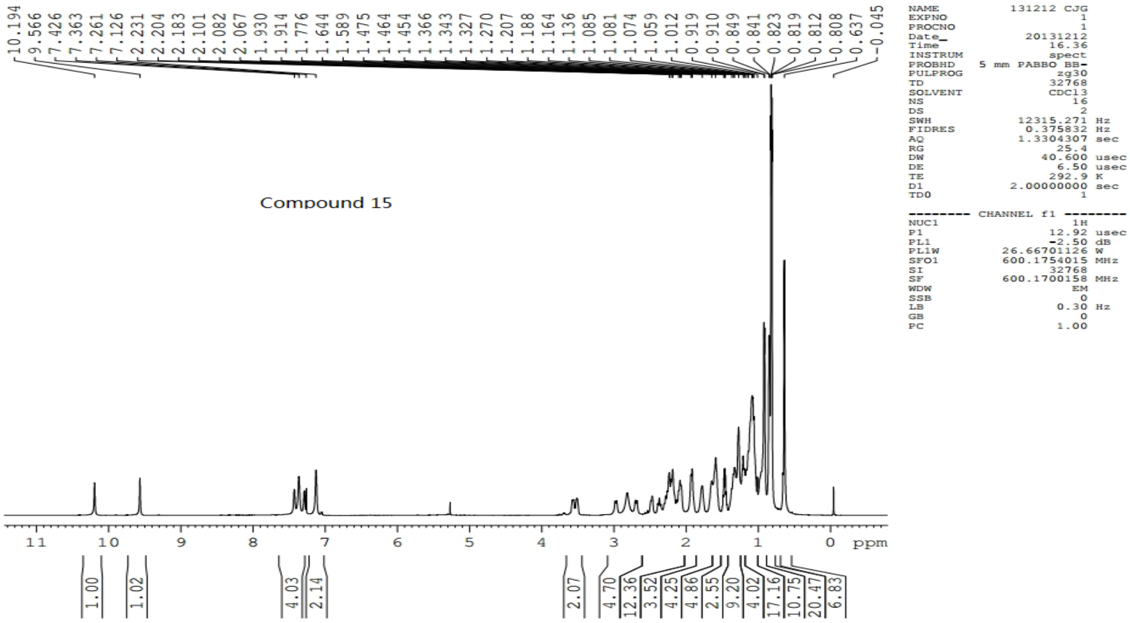

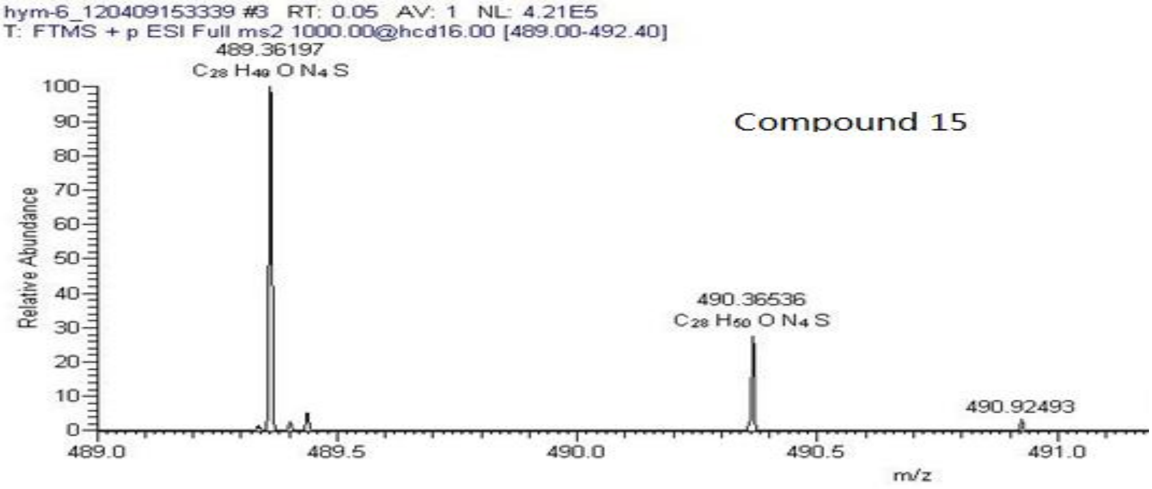

Figure S11. The NMR spectrum of 3-acetoxy-7a-aza-*B*-homocholest-5-en-7-one (**17**).

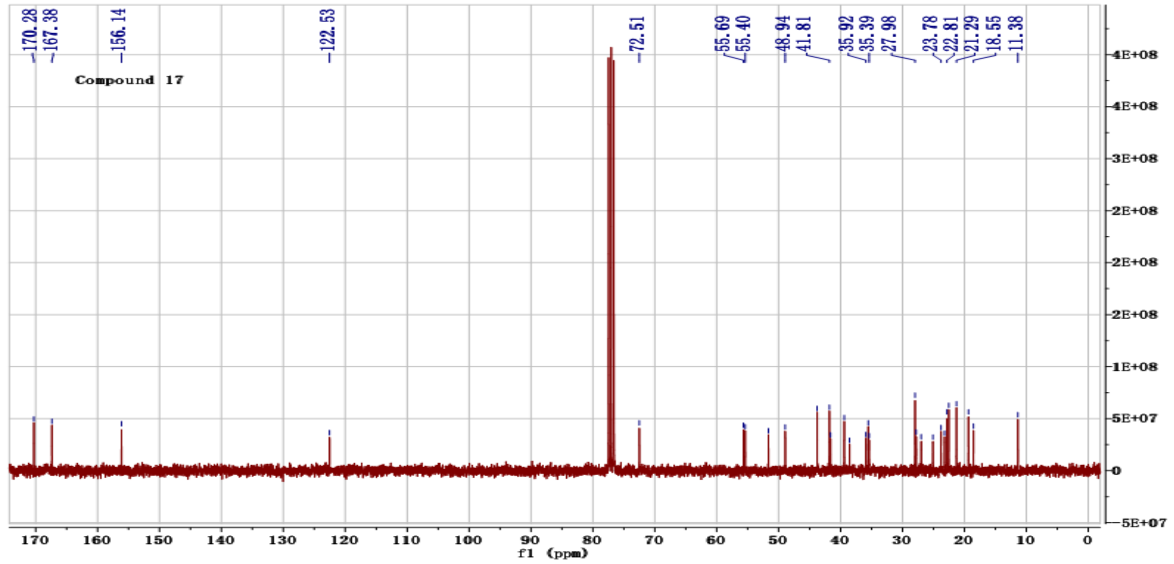

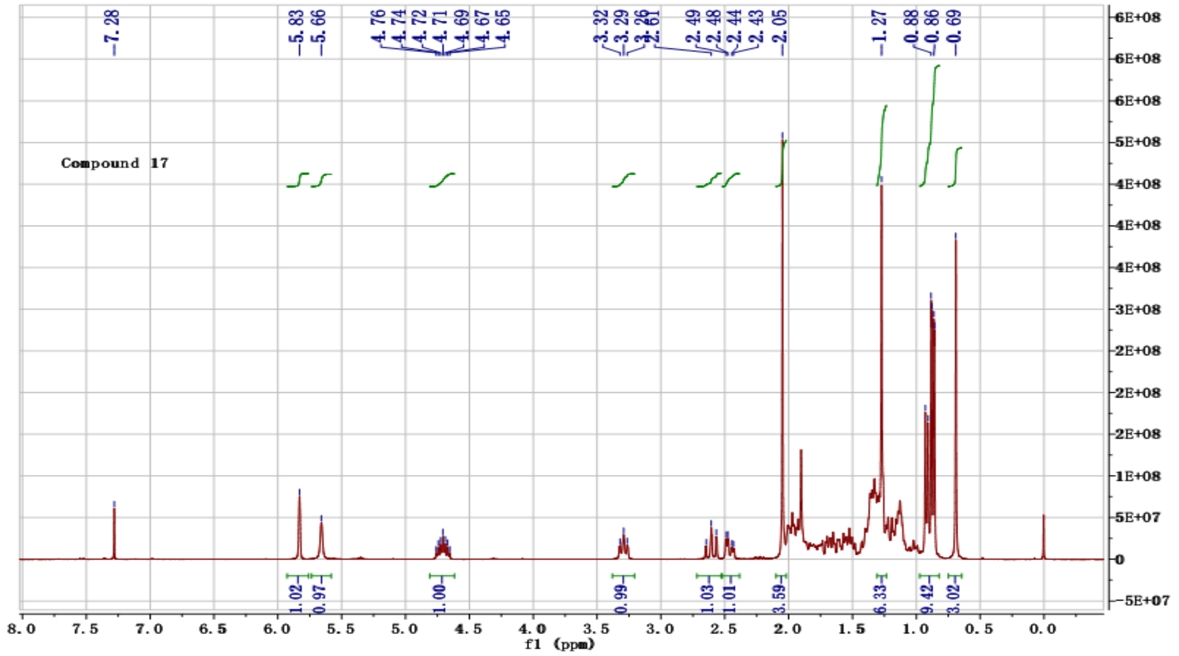

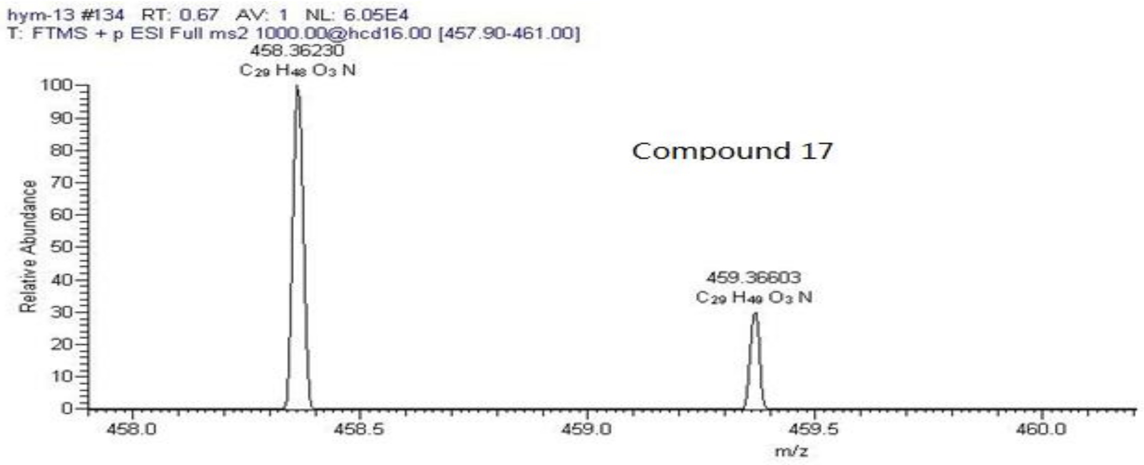

Figure S12. The NMR spectrum of 3-hydroxy-7a-aza-*B*-homocholest-5-en-7-one (**18**).

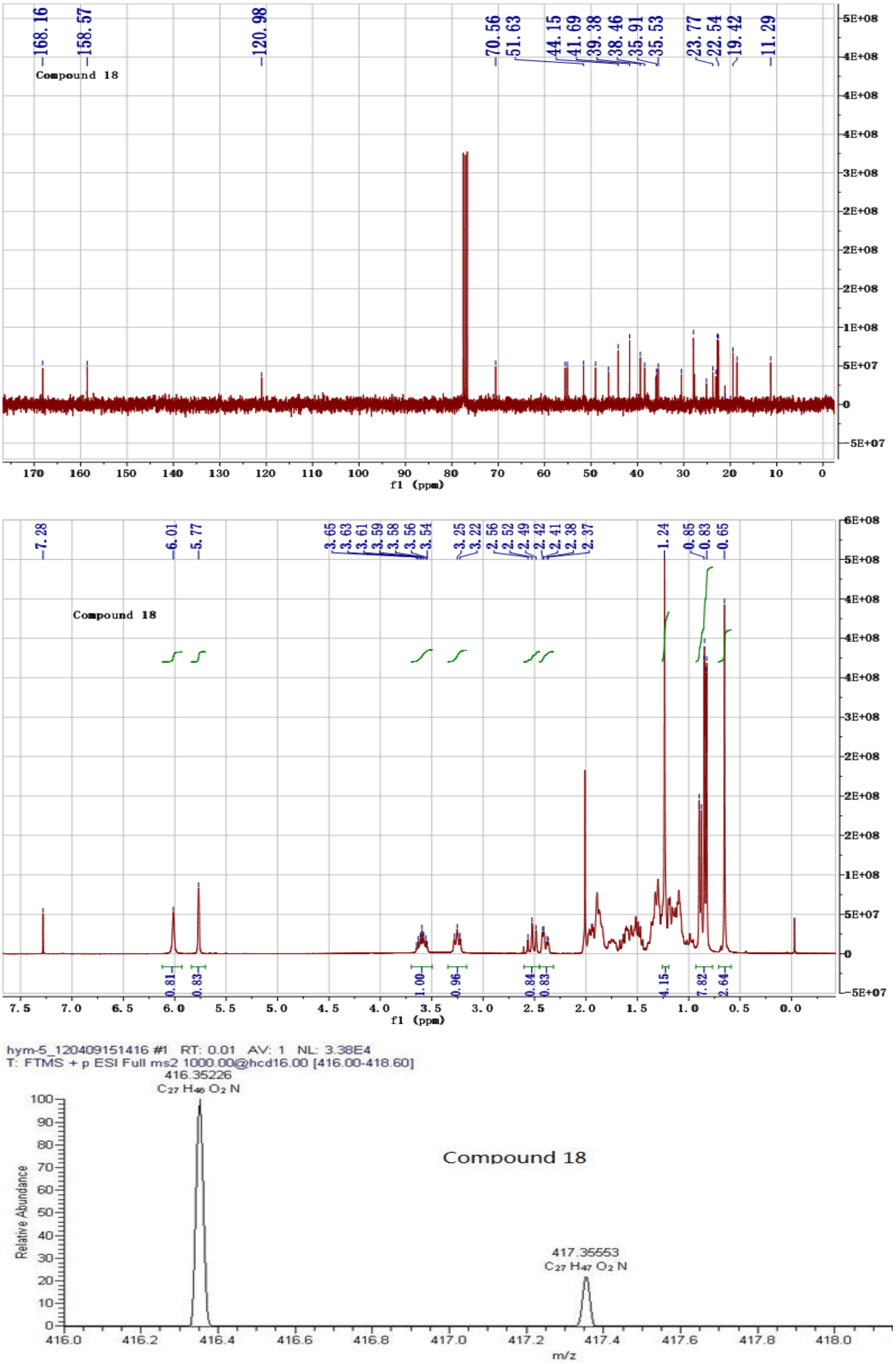

Figure S13. The NMR spectrum of 7a-aza-*B*-homocholest-4-en-3,7-dione (**19**).

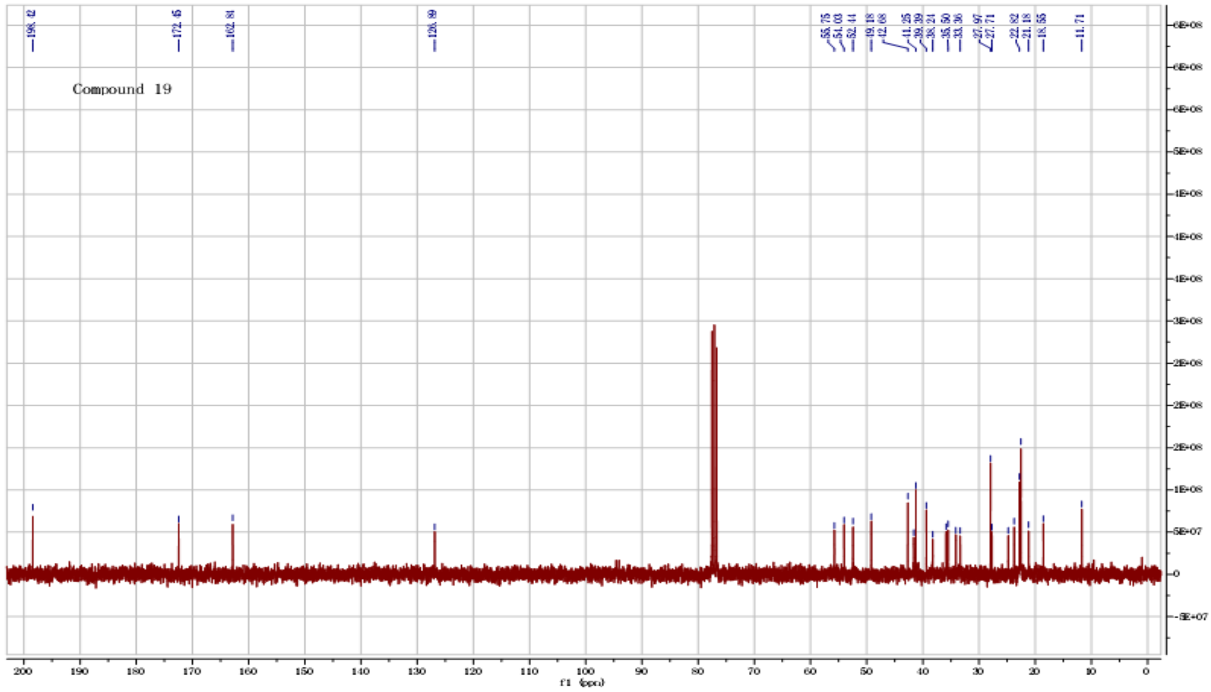

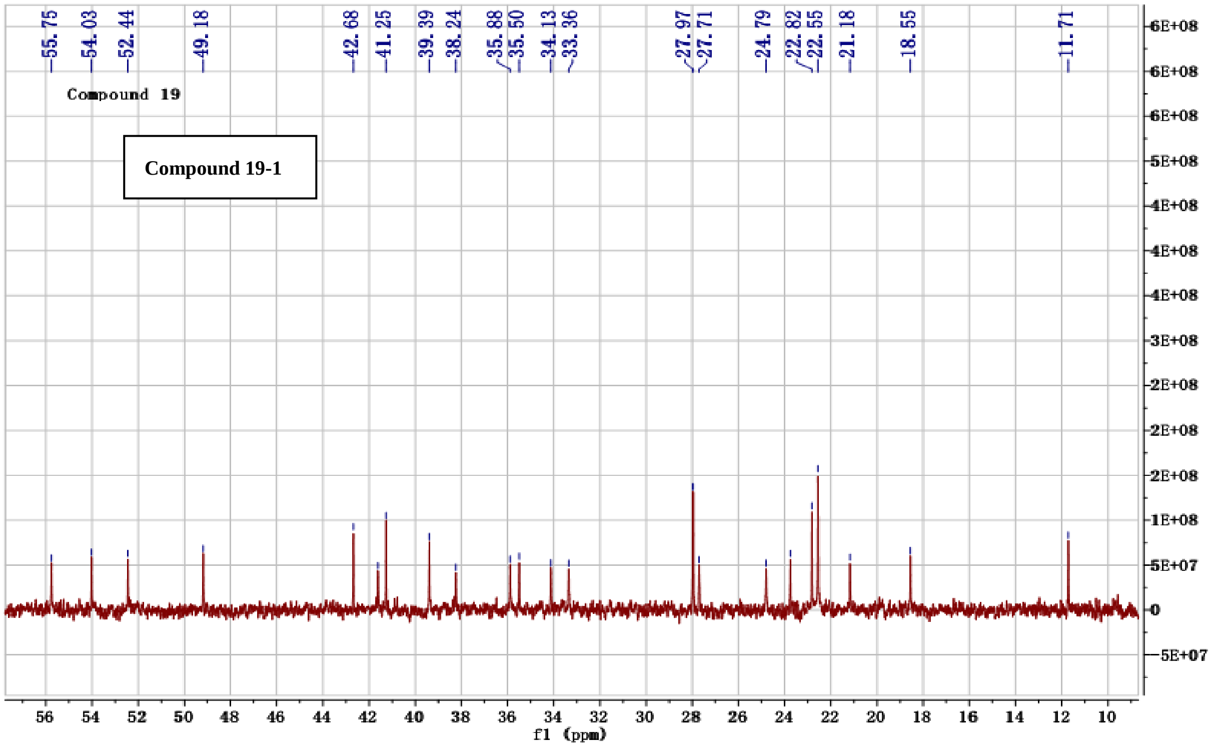

Figure S13. *Cont.*

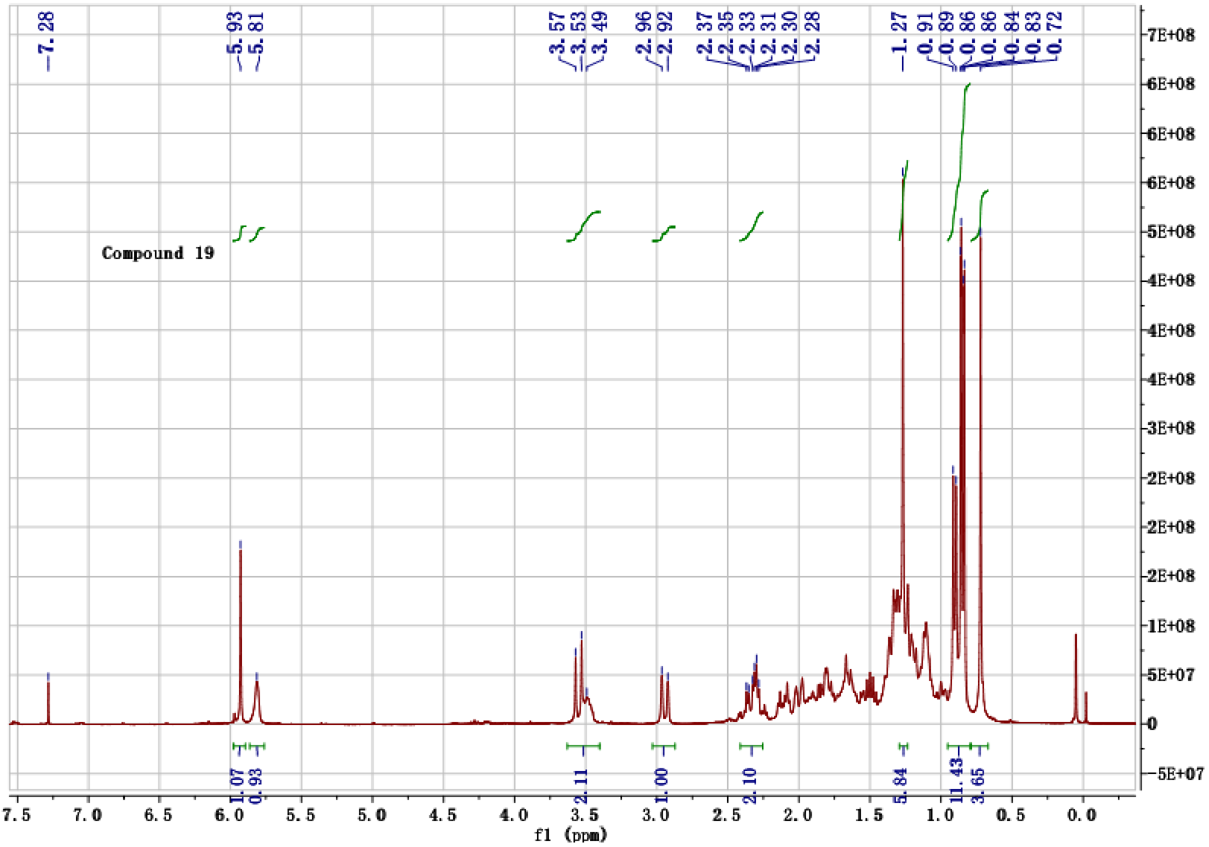

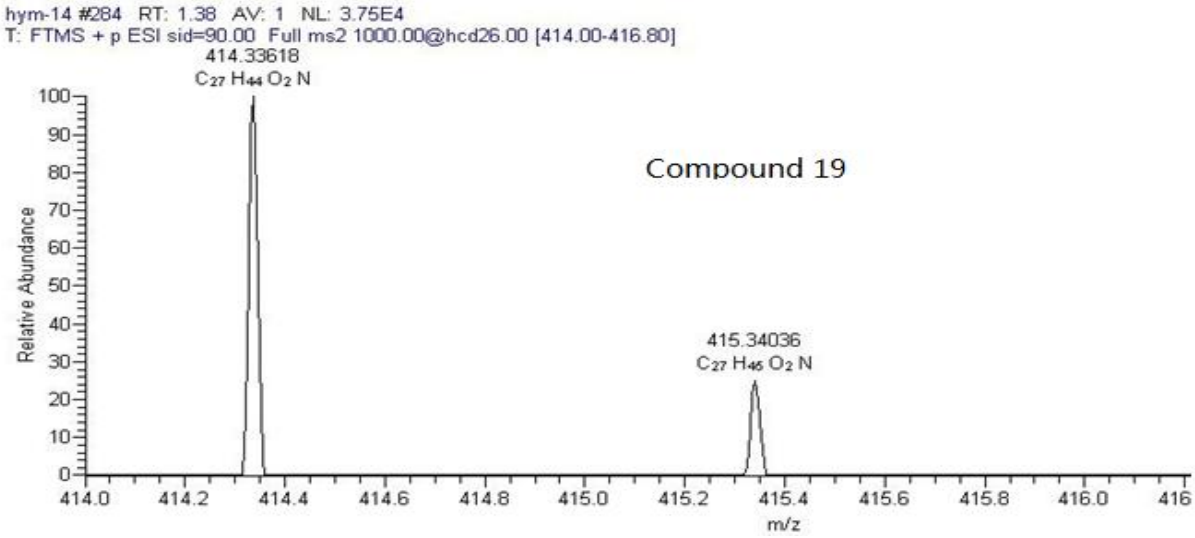

Figure S14. The NMR spectrum of 7-oxo-7a-aza-*B*-homocholest-4-en-3-thiosemicarbazone (**21**).

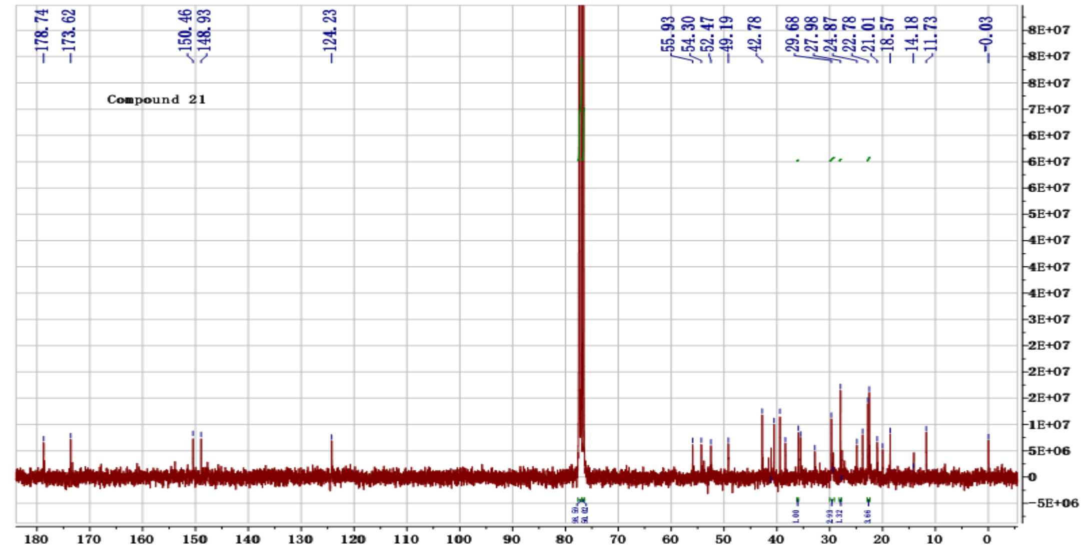

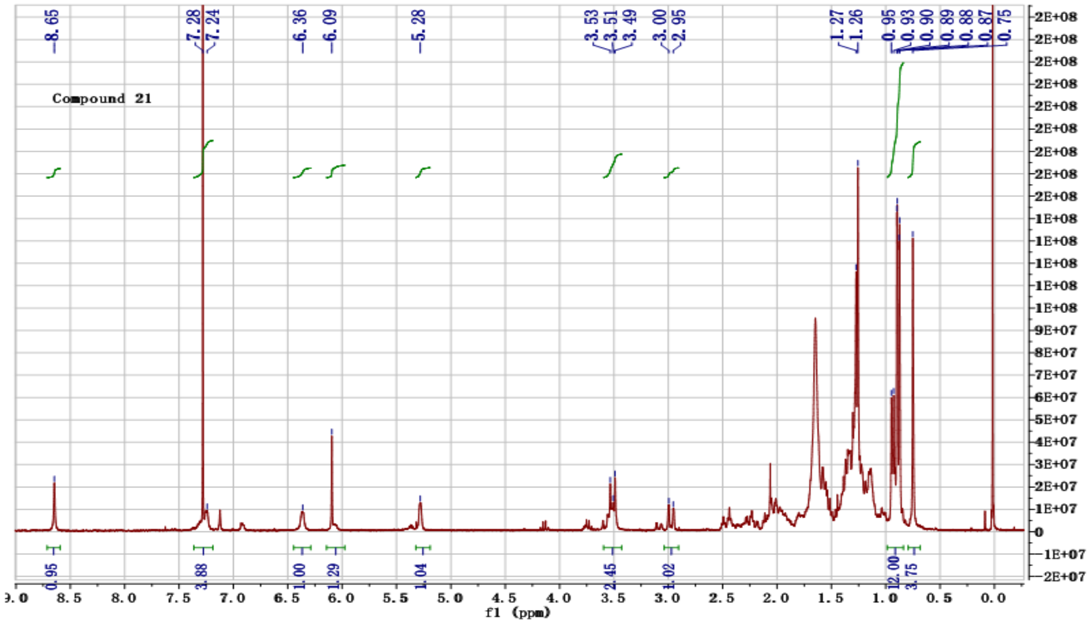

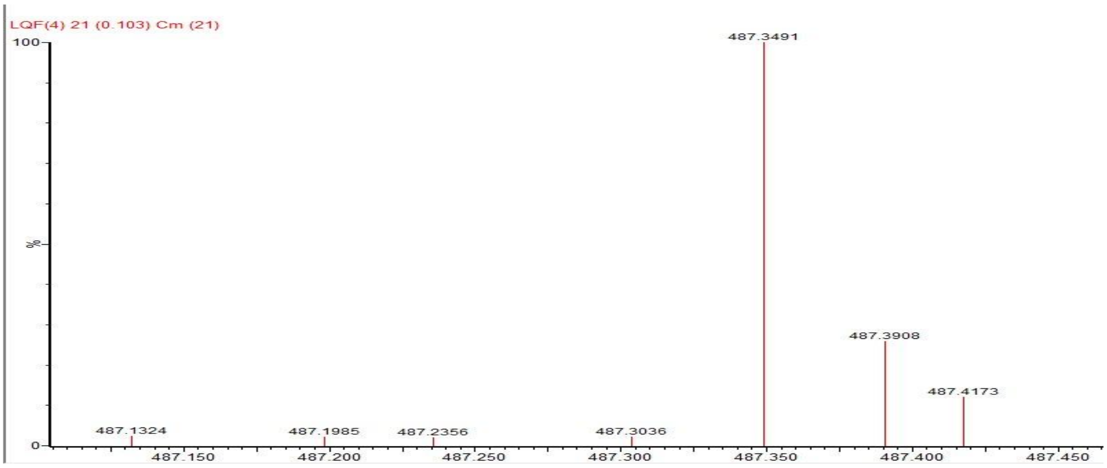

© 2014 by the authors; licensee MDPI, Basel, Switzerland. This article is an open access article distributed under the terms and conditions of the Creative Commons Attribution license (http://creativecommons.org/licenses/by/3.0/).
